# Fecal microbiota transplantation from gestational diabetes mellitus patients induces glucose intolerance and subclinical inflammation in mice

**DOI:** 10.3389/fmicb.2025.1723816

**Published:** 2026-01-28

**Authors:** Junhua Huang, Xin Yin, Yujie Zhang, Wei Zheng, Guanghui Li

**Affiliations:** Department of Nutrition, Endocrinology, and Metabolism, Beijing Obstetrics and Gynecology Hospital, Capital Medical University, Beijing Maternal and Child Health Care Hospital, Beijing, China

**Keywords:** fecal microbiota transplantation, gestational diabetes mellitus, glucose intolerance, inflammation, mouse model

## Abstract

**Background:**

The pathogenesis of gestational diabetes mellitus (GDM) is not fully understood, with gut microbiota dysbiosis emerging as a potential contributing factor. Existing animal models primarily mimic type 1 or type 2 diabetes, inadequately representing GDM. This study aimed to investigate whether fecal microbiota transplantation (FMT) from GDM patients is associated with the development of GDM-like phenotypes in mice, comparing this approach to traditional modeling methods.

**Methods:**

Fecal microbiota from GDM patients and healthy controls were transplanted into antibiotic-treated pregnant mice, creating trimester-specific FMT models. Control groups included mice on a high-fat diet (HFD) and HFD combined with streptozotocin (STZ). Metabolic phenotypes were assessed via glucose and insulin tolerance tests, fasting blood glucose, and insulin measurements. Serum inflammatory markers were analyzed, and gut inflammation was evaluated. 16S rRNA sequencing was performed on key model groups.

**Results:**

Mice receiving FMT from mid-late trimester GDM donors or traditional treatments developed significant glucose intolerance, insulin resistance, and gestational weight gain. Serum levels of inflammatory factors (e.g., IL-1β, MMP-9) were elevated. 16S rRNA sequencing revealed markedly reduced gut microbiota diversity and increased Firmicutes/Bacteroidota ratio in both GDM-FMT and traditional model groups, with similar microbial community structures and alterations in metabolic and inflammation-related pathways.

**Conclusion:**

Gut microbiota from GDM patients may disrupt glucose homeostasis and contribute to a pro-inflammatory state during pregnancy. The GDM-FMT model effectively recapitulates key metabolic, inflammatory, and microbial dysbiosis features of GDM, providing a novel and reliable experimental tool for mechanistic studies.

## Graphical Abstract

**Figure:**
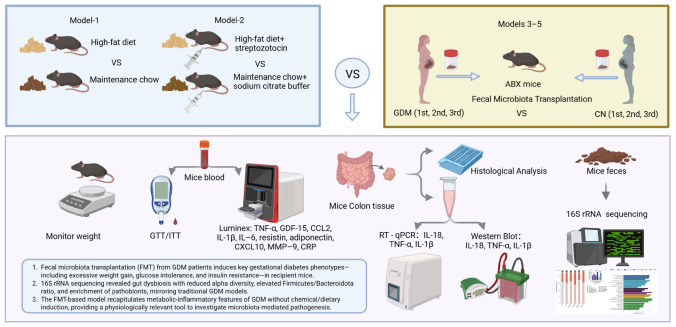
Compared with traditional methods, the FMT-induced model successfully recapitulated key features of GDM—glucose intolerance, subclinical inflammation, and gut dysbiosis—and thus serves as a viable method for GDM phenotype simulation.

## 1 Introduction

Gestational diabetes mellitus (GDM), the most prevalent metabolic disorder specific to pregnancy, affects approximately 14% of pregnancies globally (Gao et al., 2019). It is characterized by maternal hyperglycemia resulting from insufficient insulin secretion in the context of pregnancy-aggraveted insulin resistance, and poses significant acute and long-term risks to both maternal and offspring health. Immediate complications include preeclampsia, fetal macrosomia, and cesarean delivery, while long-term consequences encompass elevated risks of type 2 diabetes (T2DM) and metabolic syndrome in mothers and their offspring (Eletri and Mitanchez, 2022; Ye et al., 2022). Despite its clinical significance, the precise pathogenic mechanisms of GDM remain incompletely understood, hampering the development of effective predictive strategies and targeted early interventions.

A significant limitation in the field is the reliance on conventional experimental models that inadequately recapitulate the human GDM pathophysiology. Existing models primarily include surgical pancreatic resection, chemical β-cell ablation to induce insulin deficiency (Pasek and Gannon, 2013), and high-fat diet-induced obesity to mimic insulin resistance (Liang et al., 2010). However, these approaches often fail to capture the transient and heterogeneous nature of human GDM, particularly the complex interplay between genetic predisposition and environmental factors. Moreover, many chemically induced models, such as those using streptozotocin, produce severe and irreversible hyperglycemia that more closely resembles type 1 diabetes than the typical glucose intolerance of GDM. This gap underscores the need for more physiologically relevant models.

Emerging evidence has highlighted the association between gut microbiota dysbiosis and GDM (Koren et al., 2012; Medici Dualib et al., 2021), suggesting a potential role of the gut-microbiota-metabolite axis in the pathogenesis of GDM. The gut microbiota influences host metabolism through various mechanisms, including the production of bioactive metabolites (Wu et al., 2021; Zhu and Goodarzi, 2020). A key example involves bile acids (BAs), which are synthesized from cholesterol in hepatocytes and subsequently biotransformed by gut microbes. Dysfunctional gut microbiota can impair the conversion of primary conjugated BAs to secondary BAs in the colon, potentially by reducing bile salt hydrolase (BSH) activity. This leads to an accumulation of primary conjugated BAs and may suppress the activation of TGR5, a receptor whose activation promotes GLP-1 secretion and white adipose tissue browning—a mechanism critical for maintaining glucose homeostasis and enhancing energy expenditure (Thomas et al., 2008). Although fecal microbiota transplantation (FMT) from pregnant women to germ-free mice has been explored to understand microbial contributions to metabolic health (Liu et al., 2020), a critical limitation persists: these studies did not involve pregnant recipient mice. Since GDM is a condition exclusive to pregnancy, the absence of a pregnant state in the recipient animals means these models are not stringent GDM models. Furthermore, the comparative efficacy of this microbial-driven approach versus traditional models remains unestablished, and the mechanistic links between microbial dysbiosis and hyperglycemia require further elucidation.

Another emerging pathophysiological insight involves chronic low-grade inflammation as a potential mediator in GDM development. The pro-inflammatory state characteristic of early pregnancy appears exacerbated in individuals who develop GDM, and metabolic inflammation is hypothesized to contribute to insulin resistance (Seyhanli et al., 2022; Wang and Wang, 2020; Wolf et al., 2003). Microbial-derived metabolites, such as those involved in the tryptophan-kynurenine pathway or lipopolysaccharides (LPS), might influence this inflammatory cascade, potentially by affecting intestinal barrier integrity (Agus et al., 2021). This potential microbial-inflammation axis represents an active area of investigation, although the precise temporal sequence and molecular mechanisms are not yet fully defined.

To address these research gaps, our study introduces a novel experimental approach. We transplanted fecal microbiota from GDM patients, collected during the first, second, and third trimesters, into antibiotic-treated (ABX) mice, which were subsequently bred to pregnancy. This design fully recapitulates the GDM phenotype and allows for a direct comparison of the differential effects of microbial communities from each gestational period on GDM development. This study aims to: (1) evaluate the efficacy of this microbial-driven model in replicating key GDM phenotypes; (2) investigate the relationship between inflammatory markers and insulin resistance following FMT; and (3) perform comparative analyses with traditional chemical and dietary induction models. Our findings are expected to advance the understanding of microbial contributions to GDM pathophysiology and provide a validated experimental platform for further mechanistic investigation.

## 2 Materials and methods

### 2.1 Animals and experimental protocols

All animal procedures were approved by the Animal Ethics Committee of Beijing Huafukang Biotechnology Co., Ltd. (Approval No. HFK-AP-20230506). Healthy, pathogen-free 4-week-old female and male C57BL/6J mice were obtained from Beijing Huafukang Biotechnology Co., Ltd. (Beijing, China). After 1 week of acclimatization with standard maintenance chow (No. 1035, Beijing Huafukang Biotechnology Co., Ltd., China; Supplementary Table 1), mice were housed under controlled environmental conditions (temperature: 22–25°C; humidity: 40–60%; 12-h light/dark cycle). Female and male mice were separately caged (5 mice/cage) with *ad libitum* access to food and water. Bedding was replaced weekly. Following acclimatization, female mice were randomly assigned to experimental groups using IBM SPSS Statistics 26.0 software. Individual identification was performed via toe clipping. Five distinct animal models and their respective controls were established. Interventions commenced at 5 weeks of age. Details regarding sample size determination, randomization, and blinding procedures are provided in Supplementary Method S1.

After 4 weeks of intervention, female mice were co-housed with male mice at a 2:1 ratio for 3 consecutive days. Vaginal mucus plug detection each morning confirmed successful mating (gestational day 0.5), after which males were removed. Non-pregnant mice were excluded on the 10.5th day of pregnancy. Final pregnant mice were as follows: Model-1 (*n* = 20; HFC: *n* = 10, MC: *n* = 10), Model-2 (*n* = 17; HFC-STZ: *n* = 9, MC-SCB: *n* = 8), Model-3 (*n* = 15; GDM-1st: *n* = 8, CN-1st: *n* = 7), Model-4 (*n* = 16; GDM-2nd: *n* = 8, CN-2nd: *n* = 8), and Model-5 (*n* = 17; GDM-3rd: *n* = 9, CN-3rd: *n* = 8). Pre-pregnancy interventions were maintained throughout gestation.

To robustly evaluate the efficacy of our novel FMT-based GDM model, we selected two well-established chemical and dietary models as comparators: the high-fat diet (HFD) model and the high-fat diet combined with low-dose streptozotocin (HFD/STZ) model. These models were chosen for their distinct yet complementary mechanisms of inducing metabolic dysfunction, which collectively represent key pathophysiological facets of human GDM. The HFD model primarily induces obesity and systemic insulin resistance, mirroring the pre-pregnancy metabolic state of many women who develop GDM. In contrast, the HFD/STZ model builds upon this by adding a component of STZ-induced pancreatic β-cell dysfunction, thereby more closely simulating the subsequent failure of insulin secretion that characterizes overt GDM. By juxtaposing our microbial-driven model with these established paradigms, we aim to determine not only whether the FMT model recapitulates core GDM phenotypes but also to what extent its pathophysiology aligns with (or diverges from) that induced by pure dietary obesity (HFD) or combined insulin resistance/β-cell impairment (HFD/STZ). Such a comparative analysis will provide critical insights into whether gut microbiota dysbiosis contributes to GDM through mechanisms distinct from those captured by traditional models. The specific groupings and interventions are as follows:

*Model-1 (High-Fat Diet)*: Mice received high-fat chow (HFC; No. H10141, Beijing Huafukang Biotechnology Co., Ltd., China; Supplementary Table 1) until delivery.
*Control-1*: Mice maintained on standard chow (No. 1035) under identical conditions.
*Model-2 (High-Fat Diet + Streptozotocin):* HFC-fed mice received intraperitoneal injections of streptozotocin (40 mg/kg; No. S8050, Solarbio, China) dissolved in 0.1 mmol/L sodium citrate buffer (No. BF-0071, Dingguo Changsheng, China) on gestational days 8.5, 9.5, and 10.5.
*Control-2:* Mice fed standard chow received equivalent volumes of sodium citrate buffer.
*Models 3–5 (Antibiotic Cocktail + FMT)*: FMT involving donors from the first, second, and third trimesters of GDM, subsequent to a five-antibiotic cocktail therapy.
*Controls 3–5:* Identical ABX and FMT protocols using fecal samples from healthy pregnant donors, stratified by trimester.

### 2.2 Antibiotic cocktail pretreatment protocol

To effectively deplete the indigenous gut microbiota in mice prior to FMT, we administered a broad-spectrum antibiotic cocktail via oral gavage once daily for five consecutive days. The cocktail was prepared based on the animals’ body weight and consisted of the following compounds at the specified doses: Ampicillin (100 mg/kg; No. AA022, Genview), Vancomycin (50 mg/kg; No. V8050, Solarbio), Metronidazole (100 mg/kg; No. IM0230, Solarbio), Neomycin (100 mg/kg; No. AN214, Genview), and Amphotericin B (1 mg/kg; No. AA021, Genview). This combination was selected for its broad-spectrum efficacy against bacteria (including gram-positive, gram-negative, and anaerobic species) and fungi. Following the 5-day pretreatment, antibiotics were withdrawn, and a 2-day washout period with normal drinking water was implemented to allow for antibiotic clearance. The FMT procedures were initiated immediately after this washout period. No antibiotics were administered during the subsequent gestation period.

### 2.3 Fecal preparation and standardization

Fecal samples were obtained from pre-screened human donors (42 GDM patients and 42 healthy controls, with 14 women per trimester in each group). Samples were processed after storage at -80°C. Under strict anaerobic conditions (within an anaerobic chamber with an atmosphere of 85% N_2_, 10% H_2_, and 5% CO_2_), samples were pooled within their respective donor group and trimester. A precise mass of 1 gram of pooled stool was homogenized in 5 mL of pre-reduced, sterile phosphate-buffered saline (PBS, No. BF0011, Beijing Dingguo Changsheng Biotechnology Co., Ltd., China), achieving a 1:5 (weight/volume) ratio. The mixture was vortexed thoroughly and allowed to settle for 10 min. The resulting supernatant was then passed through a sterile 100-micron cell strainer to remove large particles, creating a smooth bacterial suspension for gavage. The fecal inoculum was primarily standardized based on the mass of the starting stool material. This well-established and practical method ensures consistent microbial input derived from a defined amount of donor fecal matter.

### 2.4 FMT administration

The prepared fecal supernatant was administered to recipient mice via oral gavage. Each mouse received a volume of 200 μL of the inoculum per session. The administration schedule was strictly followed as outlined in the protocol: FMT was performed once daily for three consecutive weeks prior to mating. Following successful mating and throughout pregnancy, the FMT administration continued every 3 days until delivery to sustain the transplanted microbiota.

### 2.5 FMT fecal donor collection and sample preparation

The FMT donors were selected from the Beijing Birth Cohort (July 1, 2021-October 30, 2022; registration No. ChiCTR220058395), which includes 14,832 individuals. This study was approved by the Ethics Committee of Beijing Obstetrics and Gynecology Hospital (Approval No. 2018-key-009-01), and informed consent was been obtained. Donor inclusion criteria were: (1) Singleton pregnant women aged between 18 and 40 years; (2) Naturally conceived and full-term delivery; (3) Complete medical records, regular prenatal check-ups, and delivery at Beijing Obstetrics and Gynecology Hospital. The exclusion criteria were strictly defined to minimize confounding factors: (1) Conception by in vitro fertilization embryo transfer; (2) Pre-existing conditions including gestational hypertension, pre-gestational diabetes or diabetic ketoacidosis, hyperlipidemia, thyroid disease, severe hepatic or renal dysfunction, cardiovascular disease, gastrointestinal diseases (e.g., constipation, diarrhea, intestinal flora dysbiosis), infectious diseases (e.g., hepatitis, tuberculosis), fever, polycystic ovary syndrome, tumors or cysts, other infections, anemia, emaciation, deafness, or history of organ surgery; (3) Pregnancy complications such as premature rupture of membranes, placenta accreta, placenta previa, or other placental abnormalities; (4) Use of insulin during pregnancy; (5) Use of probiotics, microecological agents, or other medications that may affect gut microbiota; (6) Antibiotic use within the past 3 months. The comparison of baseline characteristics among FMT donors is provided in Supplementary Table 2.

Stool samples were collected from the pregnant women during their first (8–12 gestational weeks), second (24–28 gestational weeks), and third trimesters (30–34 gestational weeks) of pregnancy. A total of 42 GDM and 42 healthy controls were included, with 14 women per trimester in each group. All samples were immediately stored at -80°C refrigerator until they were used in subsequent experiments.

At 24-28 weeks of gestation, a 75-g oral glucose tolerance test (OGTT) was conducted. Based on the test results, the subjects were classified into two groups: a gestational diabetes mellitus (GDM) group and a normal control group. GDM was diagnosed when any of the following values reached or exceeded the cut—off levels: 5.1 mmol/L for fasting blood glucose, 10.0 mmol/L for 1-h post—glucose ingestion, and 8.5 mmol/L for 2-h post—glucose ingestion.

### 2.6 Fasting blood glucose and fasting insulin measurements

On the 18.5th day of gestation, after 12 h of free access to water but fasting, the tails of the mice were disinfected with 75% medical alcohol. A small incision (0.5 mm) was made on the tail, and the first drop of blood was wiped off. Then, the blood was allowed to drip naturally onto a blood glucose test strip, and the fasting blood glucose level was detected using a Roche blood glucose meter. Subsequently, orbital blood was collected from the mice to measure the fasting insulin levels. The fasting serum insulin (FINS) was measured using a mouse ultrasensitive insulin ELISA kit (No. PI602, Beyotime Biotech. Inc., China). Based on the measured fasting blood glucose (FBG) and FINS, insulin sensitivity and islet β-cell function were evaluated using the following formulas:

HOMA-IR = (FBG × FINS)/22.5

HOMA-β% = (20 × FINS)/(FBG-3.5)

### 2.7 Glucose tolerance test and insulin tolerance test

On the 13.5th day of gestation, the mice were placed in a clean cage, allowed to drink water, and fasted for 6 h. To measure the fasting blood glucose levels, the tails of the mice were disinfected with 75% medical alcohol, a 0.5-mm section was cut off, and the first drop of blood was wiped away before measuring the glucose level. The body weight of each mouse was measured. Then, glucose (2 g/kg body weight, No. CG063, Genview, the United States) was injected intraperitoneally, and blood glucose levels were measured at 15, 30, 60, 90, and 120 min post-injection. The blood glucose levels at each time point among different groups were compared, and a blood glucose curve was plotted for each group. The area under the curve (AUC) was calculated to compare the glucose response between groups.

On the 15.5th day of gestation, after a 4-h fast, a small part of the mouse tail was disinfected and cut off. The blood glucose level was measured at 0 min, and then the mice were weighed. Subsequently, insulin (No. FI174, Genview, the United States) was injected intraperitoneally at a dose of 0.5 U/kg body weight. Blood glucose levels were measured at 15, 30, 60, 90, and 120 min after injection. A blood glucose curve was drawn, and the AUC was calculated.

### 2.8 RNA isolation and real-time quantitative polymerase chain reaction

Total RNA was extracted using Trizol reagent (No. 15596026, Invitrogen, the United States) following the manufacturer’s protocol precisely. A one-step real-time quantitative polymerase chain reaction (RT-qPCR) kit (No. AG11732, Accurate Biotechnology (human) Co., Ltd, China) was employed for reverse transcription and qPCR reactions in a single tube on an ABI 7500 Real-Time PCR system (Applied Biosystems; Thermo Fisher Scientific, Inc., United States). The thermal cycling conditions were as follows: reverse transcription at 42°C for 5 min; initial denaturation at 95°C for 10 s; followed by 40 cycles of denaturation at 95°C for 5 s and annealing/extension at 60°C for 30 s. The relative gene expression levels were analyzed using the comparative 2-ΔΔCT method, and the primer sequences are presented in Supplementary Table 3.

### 2.9 Western blot

RIPA buffer (No. P0013B, Beyotime Biotech. Inc., China) and henylmethanesulfonyl fluoride (No. ST506, Beyotime Biotech. Inc., China) were used to lyse the tissues and extract proteins. The protein concentration was determined using an Enhanced BCA Protein Assay kit (No. P0009, Beyotime Biotech. Inc., China). Then, 10 and 15% SDS-PAGE (No. D1060, Beijing Solarbio Science & Technology Co., Ltd., China) were utilized for electrophoresis, and the proteins were transferred to polyvinylidene fluoride (PVDF) membranes (No. IPVH00010, EMD Millipore, United States). The membranes were blocked with 5% non-fat dry milk at room temperature for 2 h. IL-1β mouse monoclonal antibody (1:500; No. AG2258, Beyotime Biotech. Inc., China), IL-18 rabbit polyclonal antibody (1:1,000; No. AF7266, Beyotime Biotech. Inc., China), TNF-α rabbit polyclonal antibody (1:1,000; No. AF8208, Beyotime Biotech. Inc., China), and β-actin monoclonal antibody (1:5000; No. 60008, Proteintech Group, China) were added to the membranes and incubated at 4°C with gentle agitation overnight. Finally, the membranes were washed three times with Tris-buffered saline-Tween 20 (No. T1085; Beijing Solarbio Science & Technology Co., Ltd., China) and incubated with horseradish peroxidase (HRP) goat anti-rabbit IgG (1:1,000; No. A0208, Beyotime Biotech. Inc., China) and HRP goat anti-mouse IgG (1:1,000; No. A0216, Beyotime Biotech. Inc., China) for 1 h with gentle agitation at room temperature. Immunoreactive bands were detected using Chemiluminescent HRP Substrate (No. WBKLS0500, EMD Millipore, United States). ChemiDoc TM XRS+ and Image Lab software 3.0 (Bio-Rad Laboratories, Inc., United States) were used to collect imaging information.

### 2.10 Histological analysis

Colon tissues were fixed overnight by immersion in 4% paraformaldehyde, then dehydrated in ethanol, made transparent in xylene, and embedded in paraffin to prepare 5-μm-thick sections. After hematoxylin and eosin (H&E) staining, the paraffin sections were observed under a light microscope. The scoring was performed by two independent investigators who were blinded to the experimental group assignments of all slides. The following criteria were applied:

(1) Inflammation Severity and Extent: Scored based on the degree of inflammatory cell infiltration (neutrophils, lymphocytes, plasma cells) and its depth (mucosa, submucosa, muscularis propria).

(2) Crypt Damage: Assessed according to the presence of crypt distortion, dropout, abscess formation, and goblet cell depletion. A semi-quantitative scoring system (e.g., on a scale of 0–3 for each parameter) was used, and the final score for each colon sample represented the consensus between the two pathologists. In cases of initial discrepancy, a third senior pathologist was consulted to reach a definitive score.

### 2.11 Luminex

According to the manufacturer’s instructions, the Luminex Mouse Discovery Assay (10-Plex) (No. LXSAMSM-10, Bio-Techne R&D sysems, No. United States) was used to detect inflammatory factors in serum samples from FMT model mice. The assay was capable of detecting tumor necrosis factor—alpha (TNF-α), growth differentiation factor 15 (GDF-15), C-C motif chemokine ligand 2 (CCL2), interleukin-1beta (IL-1β), interleukin-6 (IL-6), resistin, adiponectin, CXC chemokine ligand-10 (CXCL10), matrix metalloproteinases 9 (MMP-9), and C-reactive protein (CRP). The plate was read on a Luminex 200 instrument, and data acquisition and analysis were carried out using the Luminex xPONENT software. The sensitivity for each analyte, defined as the lowest concentration that can be reliably distinguished from zero. The assay detects concentrations across a wide range, typically spanning 3–5 orders of magnitude for each analyte. Both intra-assay (repeatability within a plate) and inter-assay (reproducibility between different plates) coefficients of variation (CV) were consistently below 10 and 12%, respectively, for all analytes measured.

### 2.12 16 S rRNA gene sequencing and bioinformatic analysis

Genomic DNA was extracted from murine fecal samples using the Tiangen Fecal Genomic DNA Extraction Kit. The V3-V4 hypervariable regions of the bacterial 16S rRNA gene were amplified with primers 341F and 806R. Sequencing libraries were prepared using the NEB Next^®^ Ultra™ DNA Library Prep Kit and paired-end sequencing (2 × 250 bp) was performed on the Illumina NovaSeq 6,000 platform. Raw reads were quality-filtered and assembled using FLASH. Chimeric sequences were removed using UCHIME against the SILVA database. Amplicon sequence variants (ASVs) were generated with DADA2 in QIIME2. Taxonomic annotation was performed using the SILVA 138 reference database. Alpha and beta diversity analyses were conducted after rarefaction to the minimum sequencing depth. Differential abundance analysis was performed using LEfSe (LDA score > 2). For more detailed information regarding the experimental steps, please refer to Supplementary Method S2.

### 2.13 Statistical analysis

The distribution of all continuous variables was assessed for normality using the Shapiro-Wilk test, and homoscedasticity was evaluated using Levene’s test. Based on these assumption tests, data were either transformed (e.g., log-transformation) or analyzed using corresponding non-parametric tests when assumptions were violated. Specific tests were applied according to the experimental design and data structure. For comparisons between two independent groups, an unpaired Student’s *t*-test was used for normally distributed data, while the Mann-Whitney Utest was applied for non-normally distributed data. For comparisons among three or more independent groups, one-way analysis of variance (ANOVA) followed by Tukey’s honestly significant difference (HSD) *post-hoc* test was used for parametric data; the Kruskal-Wallis test followed by Dunn’s *post-hoc* test with Bonferroni correction was applied for non-parametric data. For analyses involving two independent factors (e.g., treatment group and gestational trimester), a two-way ANOVA with Bonferroni’s *post-hoc* test was performed. Crucially, for longitudinal data (e.g., body weight measurements during gestation, glucose tolerance tests), a linear mixed-effects model was employed to account for repeated measures. The model treated time and treatment group as fixed effects and mouse ID as a random effect, thereby correctly addressing pseudo-replication. The false discovery rate (FDR) across all hypothesis tests was controlled using the Benjamini-Hochberg procedure. All analyses were performed using IBM SPSS Statistics (version 26.0) and GraphPad Prism (version 9.0). A two-tailed *p* < 0.05 was considered statistically significant for individual tests prior to FDR correction, and an FDR-adjusted *p* < 0.05 was deemed significant after multiple testing correction. The unit of analysis was clearly defined as the individual mouse for all endpoint measurements to ensure consistency.

## 3 Results

### 3.1 Fecal microbiota transplantation from GDM patients alters gestational weight gain and reproductive outcomes

**FIGURE 1:**
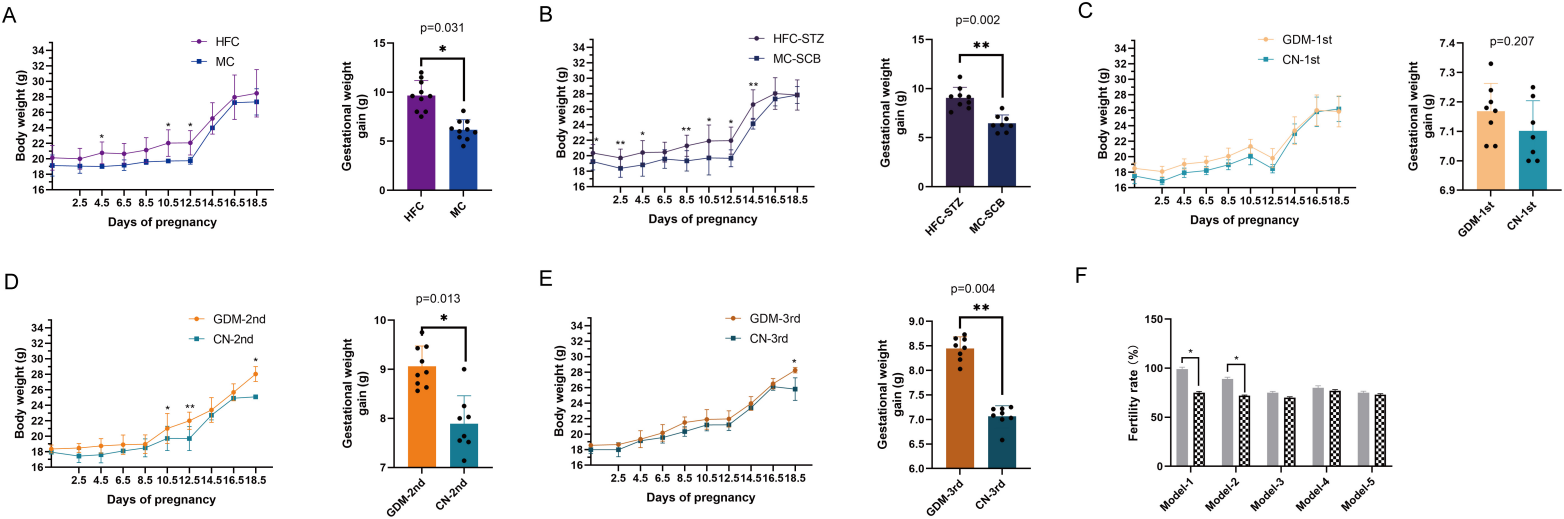
Weight and fertility rate in each model group. **(A–E)** Weight from model-1 to model-5. **(F)** Pregnancy rates for the five models. Model-1:HFC, high-fat chow; MC, maintenance chow; Model-2:HFC-STZ, high-fat chow + intraperitoneal injection of streptozotocin; MC-SCB, maintenance chow + intraperitoneal injection of sodium citrate buffer; Model-3:GDM-1st, antibiotic cocktail and fecal microbiota transplantation of the fecal in the first trimester of gestational diabetes mellitus patients; CN-1st, antibiotic cocktail and fecal microbiota transplantation of the fecal in the first trimester of health controls; Model-4:GDM-2nd, antibiotic cocktail and fecal microbiota transplantation of the fecal in the second trimester of gestational diabetes mellitus patients; CN-2nd, antibiotic cocktail and fecal microbiota transplantation of the fecal in the second trimester of health controls; Model-5: GDM-3rd, antibiotic cocktail and fecal microbiota transplantation of the fecal in the third trimester of gestational diabetes mellitus patients; CN-3rd, antibiotic cocktail and fecal microbiota transplantation of the fecal in the third trimester of health controls. Data are shown as median along with the 95% confidence intervals. Model-1 (*n* = 20; HFC: *n* = 10, MC: *n* = 10), Model-2 (*n* = 17; HFC-STZ: *n* = 9, MC-SCB: *n* = 8), Model-3 (*n* = 15; GDM-1st: *n* = 8, CN-1st: *n* = 7), Model-4 (*n* = 16; GDM-2nd: *n* = 8, CN-2nd: *n* = 8), Model-5 (*n* = 17; GDM-3rd: *n* = 9, CN-3rd: *n* = 8). *p < 0.05, **p < 0.01.

During pregnancy monitoring, we observed differential weight changes across experimental models. Mice receiving fecal microbiota transplants (FMT) from second trimester and third trimester GDM patients exhibited significantly greater gestational weight gain compared to controls. Similar patterns were noted in model-1 and model-2 groups. Notably, model-3 showed no significant weight difference (Figures 1A–E).

Reproductive assessment through vaginal plug confirmation revealed elevated pregnancy rates in high-fat diet models compared to standard chow controls. FMT models (Models 3–5) maintained comparable pregnancy rates to control groups (Figure 1F).

### 3.2 Gut microbiota modulation impacts glucose homeostasis and insulin sensitivity

**FIGURE 2:**
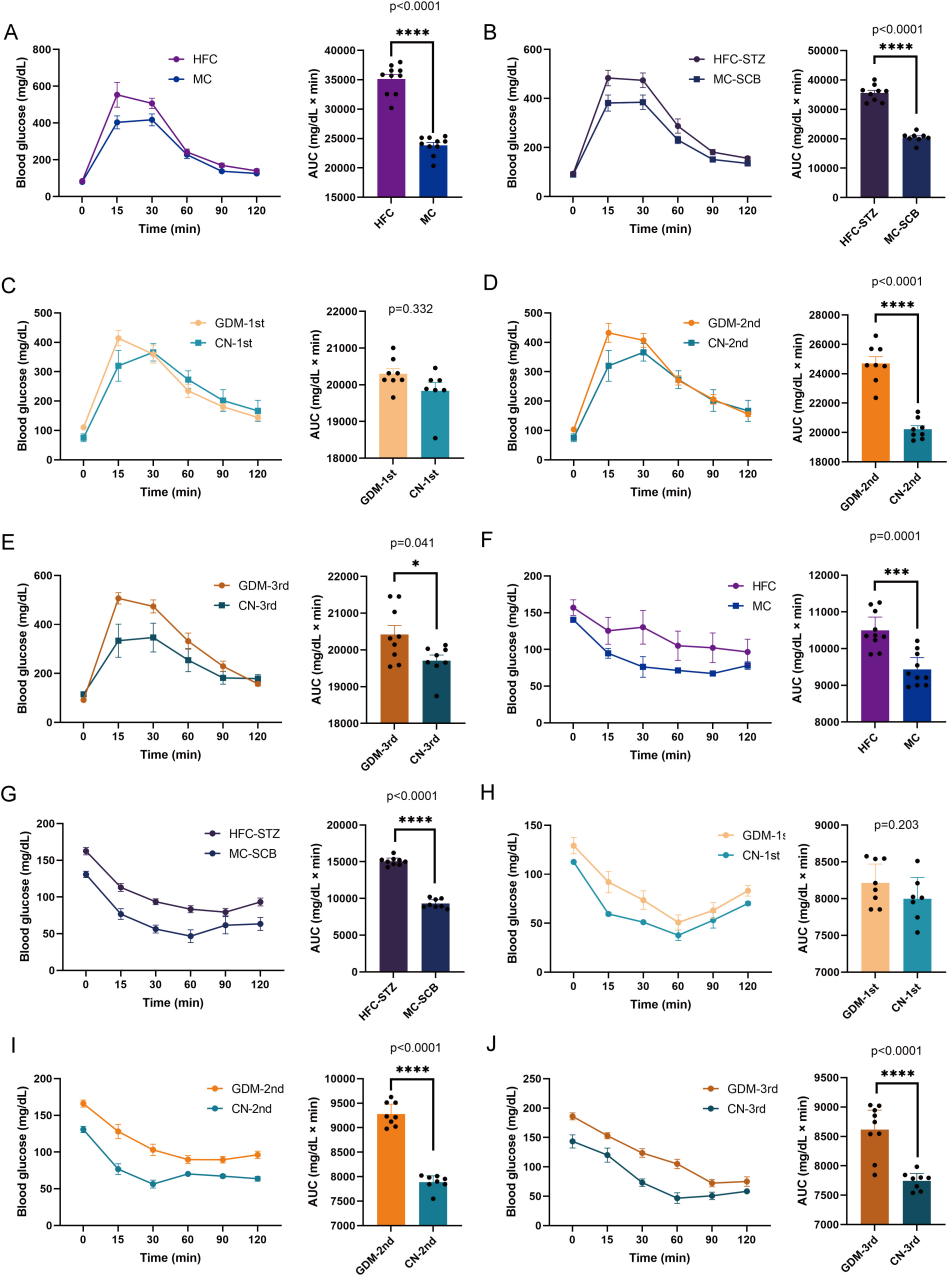
Glucose tolerance test (GTT) and insulin tolerance test (ITT) results of mice in each model group. **(A–E)** The GTT results of model-1 to model-5. **(E–J)** The ITT results of model-1 to model-5. Glucose: 2 g/kg body weight, intraperitoneal. Insulin, 0.5 U/kg body weight, intraperitoneal. The GTT was performed on the 13.5th day of gestation following a 6-h fast. The ITT was performed on the 15.5th day of gestation after a 4-h fast. Model-1:HFC, high-fat chow; MC, maintenance chow; Model-2:HFC-STZ, high-fat chow + intraperitoneal injection of streptozotocin; MC-SCB, maintenance chow + intraperitoneal injection of sodium citrate buffer; Model-3:GDM-1st, antibiotic cocktail and fecal microbiota transplantation of the fecal in the first trimester of gestational diabetes mellitus patients; CN-1st, antibiotic cocktail and fecal microbiota transplantation of the fecal in the first trimester of health controls; Model-4:GDM-2nd, antibiotic cocktail and fecal microbiota transplantation of the fecal in the second trimester of gestational diabetes mellitus patients; CN-2nd, antibiotic cocktail and fecal microbiota transplantation of the fecal in the second trimester of health controls; Model-5: GDM-3rd, antibiotic cocktail and fecal microbiota transplantation of the fecal in the third trimester of gestational diabetes mellitus patients; CN-3rd, antibiotic cocktail and fecal microbiota transplantation of the fecal in the third trimester of health controls. Data are shown as median along with the 95% confidence intervals. Model-1 (*n* = 20; HFC: *n* = 10, MC: *n* = 10), Model-2 (*n* = 17; HFC-STZ: *n* = 9, MC-SCB: *n* = 8), Model-3 (*n* = 15; GDM-1st: *n* = 8, CN-1st: *n* = 7), Model-4 (*n* = 16; GDM-2nd: *n* = 8, CN-2nd: *n* = 8), Model-5 (*n* = 17; GDM-3rd: *n* = 9, CN-3rd: *n* = 8). *p < 0.05.

GTT demonstrated impaired glycemic control in FMT recipients, particularly in second/third trimester GDM-FMT groups. High-fat diet models similarly exhibited elevated glucose excursion versus controls (Figures 2A–E). ITT revealed persistent hyperglycemia in HFC and HFC-STZ groups versus controls. GDM-2nd FMT mice showed comparable insulin resistance to HFC models (Figures 2F–J).

**FIGURE 3:**
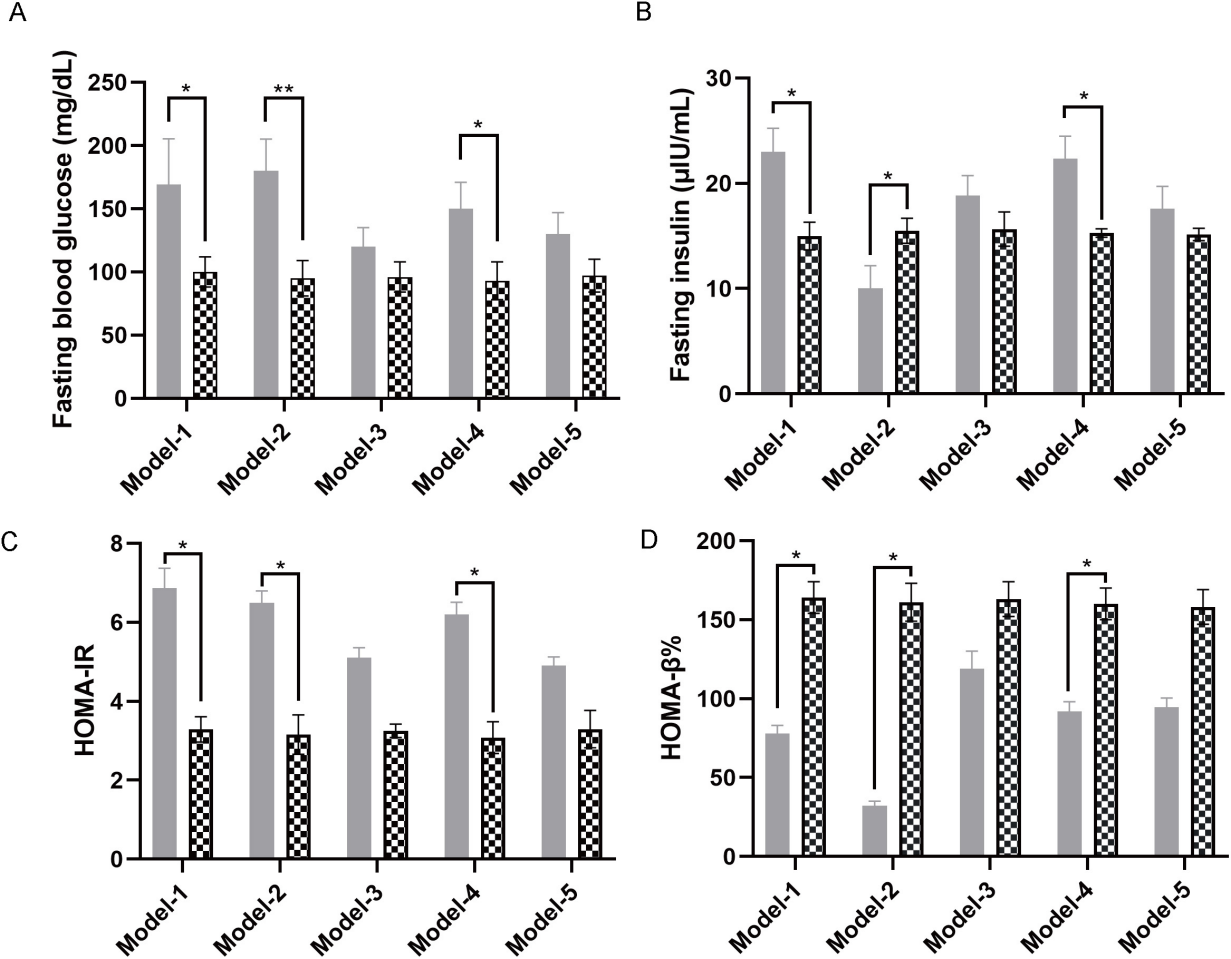
Glycemic traits of the five models. **(A)** Fasting blood glucose of five models. **(B)** Fasting insulin of five models. **(C,D)** HOMA-β% and HOMA-IR. HOMA-IR = (FBG × FINS)/22.5, HOMA-β% = (20 × FINS)/(FBG-3.5). Model-1 (*n* = 20; HFC: *n* = 10, MC: *n* = 10), Model-2 (*n* = 17; HFC-STZ: *n* = 9, MC-SCB: *n* = 8), Model-3 (*n* = 15; GDM-1st: *n* = 8, CN-1st: *n* = 7), Model-4 (*n* = 16; GDM-2nd: *n* = 8, CN-2nd: *n* = 8), Model-5 (*n* = 17; GDM-3rd: *n* = 9, CN-3rd: *n* = 8). Data are shown as mean ± SEM. *p < 0.05, **p < 0.01.

Metabolic profiling showed elevated fasting glucose in models 1 and 4. Paradoxical insulin dynamics emerged with hyperinsulinemia in models 1 and 4, contrasted by hypoinsulinemia in model-2. Corresponding HOMA-IR elevations and β-cell function decline confirmed metabolic dysregulation (Figure 3).

### 3.3 Systemic and intestinal immune signatures of microbiota perturbation

We collected the orbital blood of mice and used the luminex technology to detect the levels of 10 inflammatory factors in their serum. These factors included tumor necrosis factor-alpha (TNF-α), growth differentiation factor 15 (GDF-15), C-C motif chemokine ligand 2 (CCL2), and interleukin-1beta (IL-1β), interleukin-6 (IL-6), Resistin, adiponectin, CXC chemokine ligand-10 (CXCL10), matrix metalloproteinases 9 (MMP-9), and C-Reactive Protein (CRP). Unfortunately, we could not detect the actual concentration of CXCL10 because its concentration in each sample was too low, and the fluorescence value was lower than the background wells.

In the serum of mice fed a high-fat diet in Model-1, no significant differences in factors were found between the model group and the control group after FDR correction (Table 1).

**TABLE 1:** Expression of inflammatory markers in each model-1 (pg/mL).

Markers	Model-1			*P-*value	FDR
	Overall	HFC	MC		
TNF-α	4.64 (4.50, 12.92)	4.60 (4.48, 21.00)	4.64 (4.54, 20.82)	0.41	0.56
GDF-15	43.05 (39.14, 52.62)	39.89 (13.11, 52.62)	43.35 (40.34, 62.35)	0.24	0.40
CCL2	711.19 (691.46, 2066.35)	695.40 (646.05, 819.08)	762.46 (708.22, 851.14)	0.15	0.40
IL-1β	258.24 (254.27, 752.89)	258.24 (254.27, 281.61)	256.26 (253.25, 271.12)	0.94	0.97
IL-6	23.29 (22.19, 62.45)	23.29 (19.99, 104.09)	24.39 (22.47, 104.80)	0.25	0.40
Adiponectin	171988.86 (167113.73, 175843.60)	173434.57 (169936.72, 181763.02)	168821.18 (21697.14, 172100.51)	0.03*	0.27
Resistin	6283.42 (6180.05, 6359.87)	6322.14 (6223.97, 6374.20)	6263.69 (6213.56, 6346.72)	0.57	0.67
MMP-9	12329.44 (10811.76, 22698.08)	11306.90 (11862.65, 22698.08)	14724.98 (11085.70, 21867.24)	0.20	0.40
CRP	69366.61 (63774.06, 84337.54)	74958.53 (65856.40, 93078.02)	67167.42 (51943.87, 69809.82)	0.18	0.40

*p < 0.05.

In model-2, the levels of MMP-9 were higher in the treated mice compared to the untreated mice after FDR correction [14374.61 (10125.63, 21717.34) pg/mL vs. 11513.53 (3850.29, 14521.27) pg/mL, *p* < 0.01] (Table 2).

**TABLE 2:** Expression of inflammatory markers in each model-2 (pg/mL).

Markers	Model-2			*P-*value	FDR
	Overall	HFC-STZ	MC-SCB		
TNF-α	4.72 (4.63, 21.4)	14.20 (4.68, 21.4)	9.58 (4.44, 19.72)	0.13	0.21
GDF-15	42.6 (21.24, 262.35)	20.04 (13.86, 209.64)	23.35 (10.34, 262.35)	0.06	0.18
CCL2	821.6 (705.27, 848.70)	727.58 (671.71, 848.70)	732.46 (608.23, 851.14)	0.82	0.85
IL-1β	264.19 (254.27, 1231.69)	754.88 (267.16, 1261.44)	656.26 (234.27, 1271.12)	0.04*	0.18
IL-6	24.39 (23.29, 102.71)	68.22 (23.57, 113.01)	64.39 (21.45, 124.80)	0.26	0.37
Adiponectin	172010.7 (166723.60, 178781.77)	168901.44 (19474.02, 179141.94)	171424.47 (168916.72, 182153.12)	0.11	0.21
Resistin	6241.63 (6188.90, 6345.31)	6191.22 (6188.88, 6474.26)	6222.35 (6193.36, 6336.72)	0.32	0.37
MMP-9	10965.52 (8930.64, 16312.03)	14374.61 (10125.63, 21717.34)	11513.53 (3850.29, 14521.27)	< 0.01*	<0.01*
CRP	69746.42 (61136.27, 108038.85)	90835.38 (70962.07, 116136.37)	77167.42 (51943.87, 111809.82)	0.08	0.18

*p < 0.05.

In model-3, GDF-15 [31.50 (19.10, 27.21) pg/mL vs. 25.15 (15.36, 26.20) pg/mL, *p* < 0.01] and IL-1β [491.20 (307.75, 1291.19) pg/mL vs. 266.16 (244.35 (266.17, 278.06) pg/mL, *p* < 0.01] were higher in the mice with GDM-1st FMT than the control group after FDR correction (Table 3).

**TABLE 3:** Expression of inflammatory markers in each model-3 (pg/mL).

Markers	Model-3			*P-*value	FDR
	Overall	GDM-1st	CN-1st		
TNF-α	4.64 (4.32, 21.3)	4.54 (4.16, 20.90)	4.58 (4.34, 37.77)	0.29	0.41
GDF-15	26.35 (24.78, 29.59)	31.50 (19.10, 27.21)	25.15 (15.36, 26.20)	< 0.01*	<0.01*
CCL2	695.4 (624.32, 3319.08)	695.40 (632.22, 1319.08)	721.12 (553.15, 1120.10)	1.00	1.00
IL-1β	282.02 (278.06, 491.20)	491.20 (307.75, 1291.19)	266.16 (244.35 (266.17, 278.06)	< 0.01*	<0.01*
IL-6	37.57 (28.78, 105.46)	37.57 (29.33, 102.71)	38.23 (21.64, 118.25)	1.12	1.00
Adiponectin	162462.17 (159813.00, 171356.40)	171956.85 (170561.13, 172880.56)	160020.36 (113066.98, 162304.92)	0.06	0.08
Resistin	6077.48 (5798.26, 6200.75)	6192.22 (6104.98, 6302.27)	5972.54 (5811.41, 6083.96)	0.29	0.41
MMP-9	19965.24 (18232.88, 20615.91)	18325.47 (18049.14, 35828.72)	20214.30 (19954.59, 20529.91)	0.28	0.41
CRP	71327.28 (64689.16, 75584.43)	71327.28 (66494.97, 102851.68)	58350.01 (43268.79, 75396.32)	0.32	0.41

*p < 0.05.

In model-4, we found resistin [6345.18 (6267.49, 13637.56) pg/mL vs. 5802.34 (5701.64, 8983.56) pg/mL, *p* < 0.01], and MMP-9 [32861.06 (20979.94, 67122.49) pg/mL vs. 20263.25 (19856.24, 22364.21), *p* < 0.01] were higher in the mice with GDM-2nd FMT than the control group after FDR correction (Table 4).

**TABLE 4:** Expression of inflammatory markers in each model-4 (pg/mL).

Markers	Model-4			*P-*value	FDR
	Overall	GDM-2nd	CN-2nd		
TNF-α	4.76 (4.42, 7.98)	4.76 (4.50, 7.54)	4.53 (4.34, 7.77)	0.52	0.69
GDF-15	49.98 (40.46, 593.81)	49.98 (44.07, 322.45)	51.50 (49.10, 297.83)	0.63	0.69
CCL2	1306.48 (683.52, 1534.21)	1306.48 (1139.59, 1429.24)	1039.23 (553.15, 1424.31)	0.62	0.69
IL-1β	270.13 (252.29, 280.04)	274.09 (252.29, 284.58)	266.16 (244.36, 278.06)	0.25	0.67
IL-6	35.65 (25.76, 40.19)	35.66 (32.77, 38.25)	36.21 (21.64, 38.24)	0.63	0.69
Adiponectin	172633.95 (170884.50, 175307.50)	176168.13 (168866.56, 1608064.32)	175956.85 (171561.13, 181880.56)	0.26	0.67
Resistin	6183.18 (5997.41, 9324.78)	6345.18 (6267.49, 13637.56)	5802.34 (5701.64, 8983.56)	< 0.01*	0.04*
MMP-9	20757.73 (20231.79, 26998.69)	32861.06 (20979.94, 67122.49)	20263.25 (19856.24, 22364.21)	< 0.01*	0.04*
CRP	64134.62 (57544.17, 75396.33)	64134.63 (62968.16, 71163.12)	62131.78 (58965.45, 70125.69)	0.34	0.68

*p < 0.05.

In model-5, there was no significant difference in the serum levels of nine inflammatory factors between the model and control groups (Table 5).

**TABLE 5:** Expression of inflammatory markers in each model-5 (pg/mL).

Markers	Model-5			*P-*value	FDR
	Overall	GDM-3rd	CN-3rd		
TNF-α	4.76 (4.36, 21.4)	4.75 (4.44, 21.3)	4.53 (4.34, 27.78)	0.81	0.97
GDF-15	30.41 (28.91, 69.89)	30.41 (12.01, 67.85)	31.50 (29.10, 57.83)	0.20	0.71
CCL2	703.3 (612.46, 810.71)	703.30 (620.37, 828.96)	703.54 (553.15, 798.13)	1.00	1.00
IL-1β	270.13 (254.27, 278.06)	270.13 (254.27, 275.36)	261.87 (224.36, 281.64)	0.44	0.78
IL-6	25.49 (23.01, 45.94)	25.49 (23.29, 38.56)	26.13 (21.03, 40.12)	0.77	0.97
Adiponectin	168175.39 (158645.30, 172634.00)	159398.44 (112362.54, 169998.79)	168594.65 (124412.32, 171036.52)	0.06	0.61
Resistin	6022.28 (5798.26, 6164.66)	6150.90 (6128.77, 9229.16)	5972.54 (5811.41, 6083.96)	0.48	0.78
MMP-9	19965.24 (12720.69, 20615.91)	13124.55 (11175.01, 21907.45)	10113.25 (10098.16, 20529.91)	0.28	0.71
CRP	49479.63 (42890.06, 67945.56)	49479.63 (28726.56, 66302.06)	50213.02 (25496.31, 70145.21)	0.29	0.71

**FIGURE 4:**
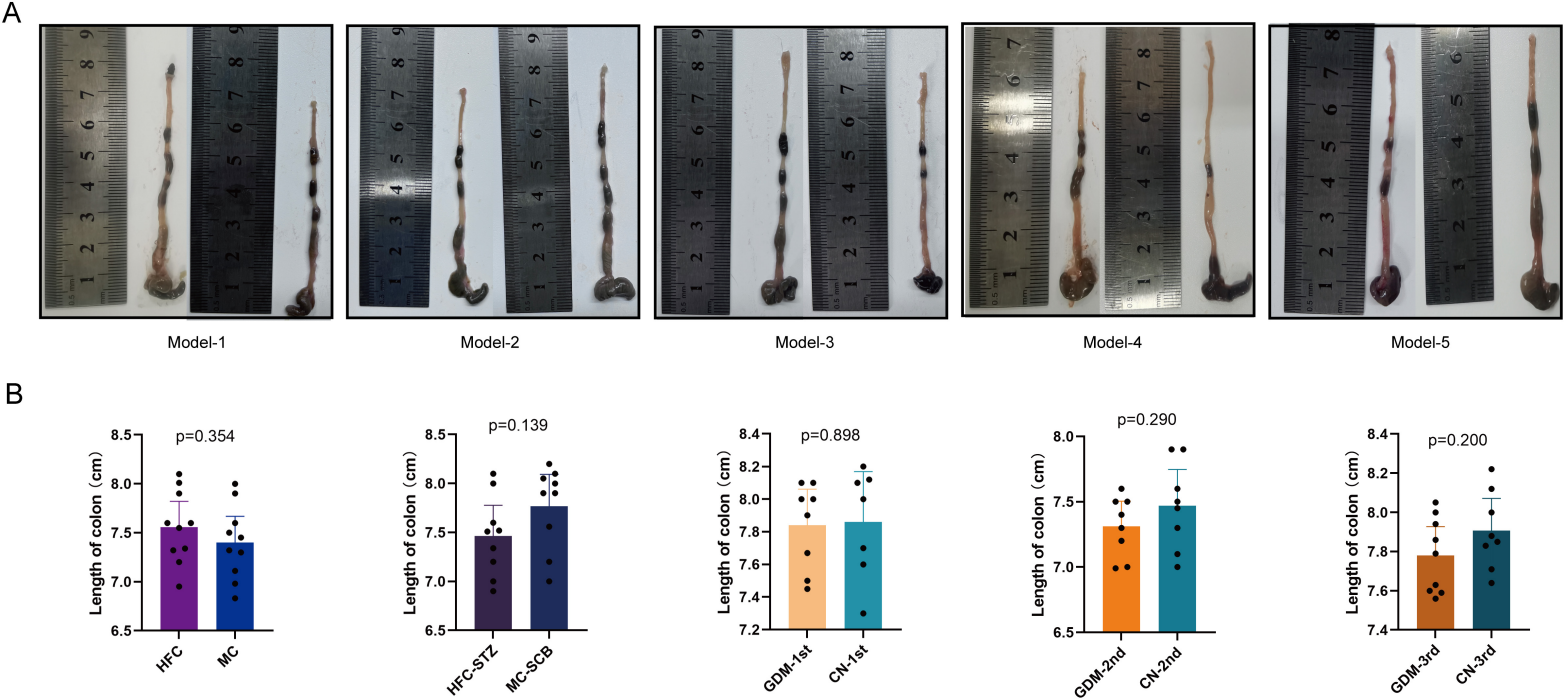
The length of colon in five models. **(A)** Photographs of the colon. **(B)** Bar graph of colon length comparison in each group. The colon was collected after euthanasia on gestational day 18.5. Model-1:HFC, high-fat chow; MC, maintenance chow; Model-2:HFC-STZ, high-fat chow + intraperitoneal injection of streptozotocin; MC-SCB, maintenance chow + intraperitoneal injection of sodium citrate buffer; Model-3:GDM-1st, antibiotic cocktail and fecal microbiota transplantation of the fecal in the first trimester of gestational diabetes mellitus patients; CN-1st, antibiotic cocktail and fecal microbiota transplantation of the fecal in the first trimester of health controls; Model-4:GDM-2nd, antibiotic cocktail and fecal microbiota transplantation of the fecal in the second trimester of gestational diabetes mellitus patients; CN-2nd, antibiotic cocktail and fecal microbiota transplantation of the fecal in the second trimester of health controls; Model-5: GDM-3rd, antibiotic cocktail and fecal microbiota transplantation of the fecal in the third trimester of gestational diabetes mellitus patients; CN-3rd, antibiotic cocktail and fecal microbiota transplantation of the fecal in the third trimester of health controls. Data are shown as median along with the 95% confidence intervals. Model-1 (*n* = 20; HFC: *n* = 10, MC: *n* = 10), Model-2 (*n* = 17; HFC-STZ: *n* = 9, MC-SCB: *n* = 8), Model-3 (*n* = 15; GDM-1st: *n* = 8, CN-1st: *n* = 7), Model-4 (*n* = 16; GDM-2nd: *n* = 8, CN-2nd: *n* = 8), Model-5 (*n* = 17; GDM-3rd: *n* = 9, CN-3rd: *n* = 8).

The length of colon did not differ between the model and control mice in any of the five models (Figure 4).

**FIGURE 5:**
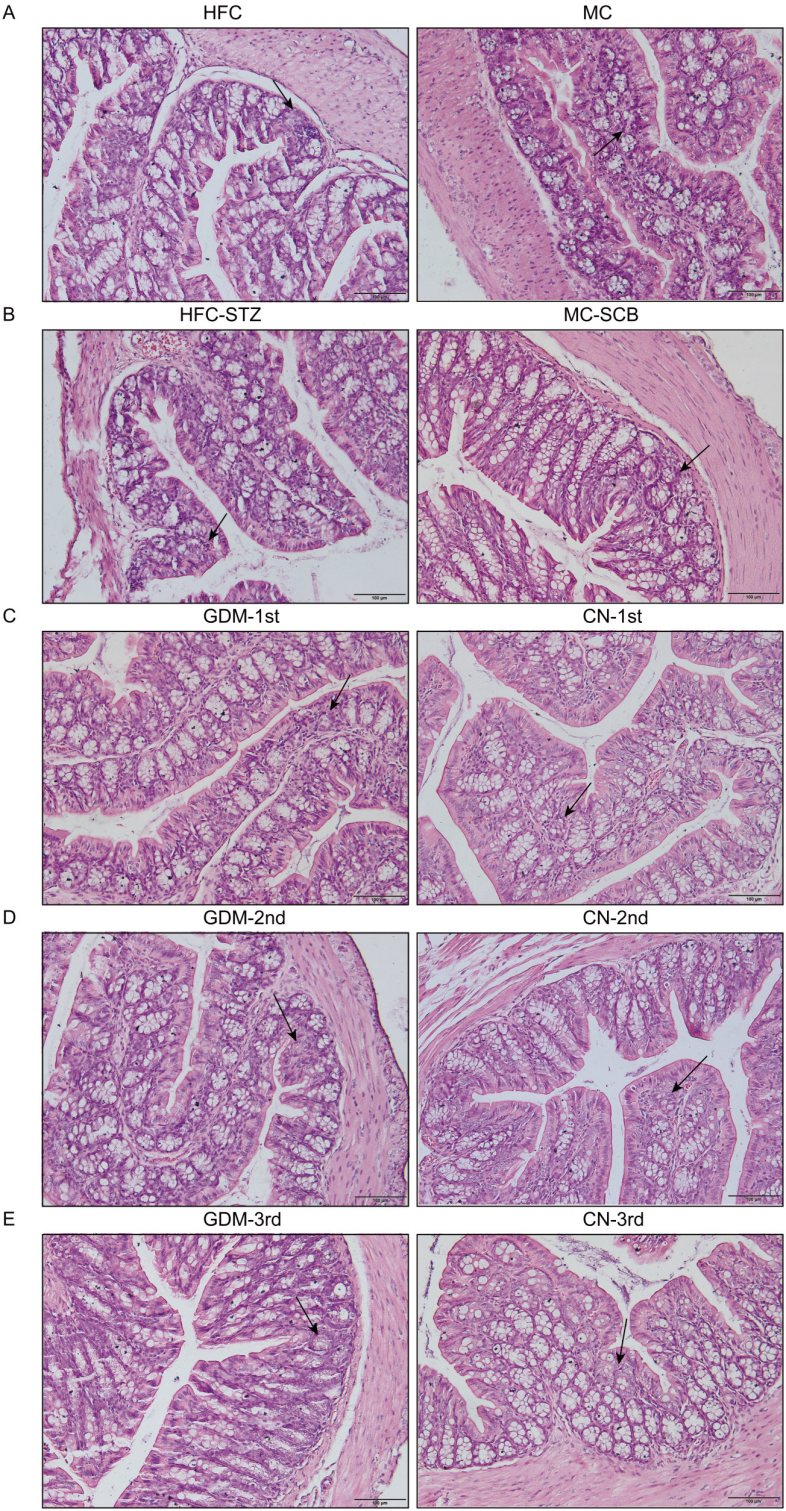
Light microscope images of colonic tissues stained with HE in each model group. The scale bar in the lower right corner represents 100 μm, 100×. Black arrow indicates the crypt and some lymphoplasmacytic cells. **(A)** Model-1:HFC, high-fat chow; MC, maintenance chow; **(B)** Model-2:HFC-STZ, high-fat chow + intraperitoneal injection of streptozotocin; MC-SCB, maintenance chow + intraperitoneal injection of sodium citrate buffer; **(C)** Model-3:GDM-1st, antibiotic cocktail and fecal microbiota transplantation of the fecal in the first trimester of gestational diabetes mellitus patients; CN-1st, antibiotic cocktail and fecal microbiota transplantation of the fecal in the first trimester of health controls; **(D)** Model-4:GDM-2nd, antibiotic cocktail and fecal microbiota transplantation of the fecal in the second trimester of gestational diabetes mellitus patients; CN-2nd, antibiotic cocktail and fecal microbiota transplantation of the fecal in the second trimester of health controls; **(E)** Model-5: GDM-3rd, antibiotic cocktail and fecal microbiota transplantation of the fecal in the third trimester of gestational diabetes mellitus patients; CN-3rd, antibiotic cocktail and fecal microbiota transplantation of the fecal in the third trimester of health controls.

After performing a HE staining on the colon tissue of each group of mice, we observed that there were no significant changes in the structure, shape, thickness, and size of the mucosa, submucosa, muscular layer, and adventitia of the intestinal tissue. The polarity of the colonic mucosal epithelium was satisfactory, and the proliferation of cryptids was not evident. However, some areas of cryptids had poor polarity. Lymphoplasmacytic cells were present in the stroma, but only a few of them. The distance between the base of the villous crypt and the myometrium was normal, as well as the myometrium and serosal layer, which were also basically normal (Figure 5).

**FIGURE 6:**
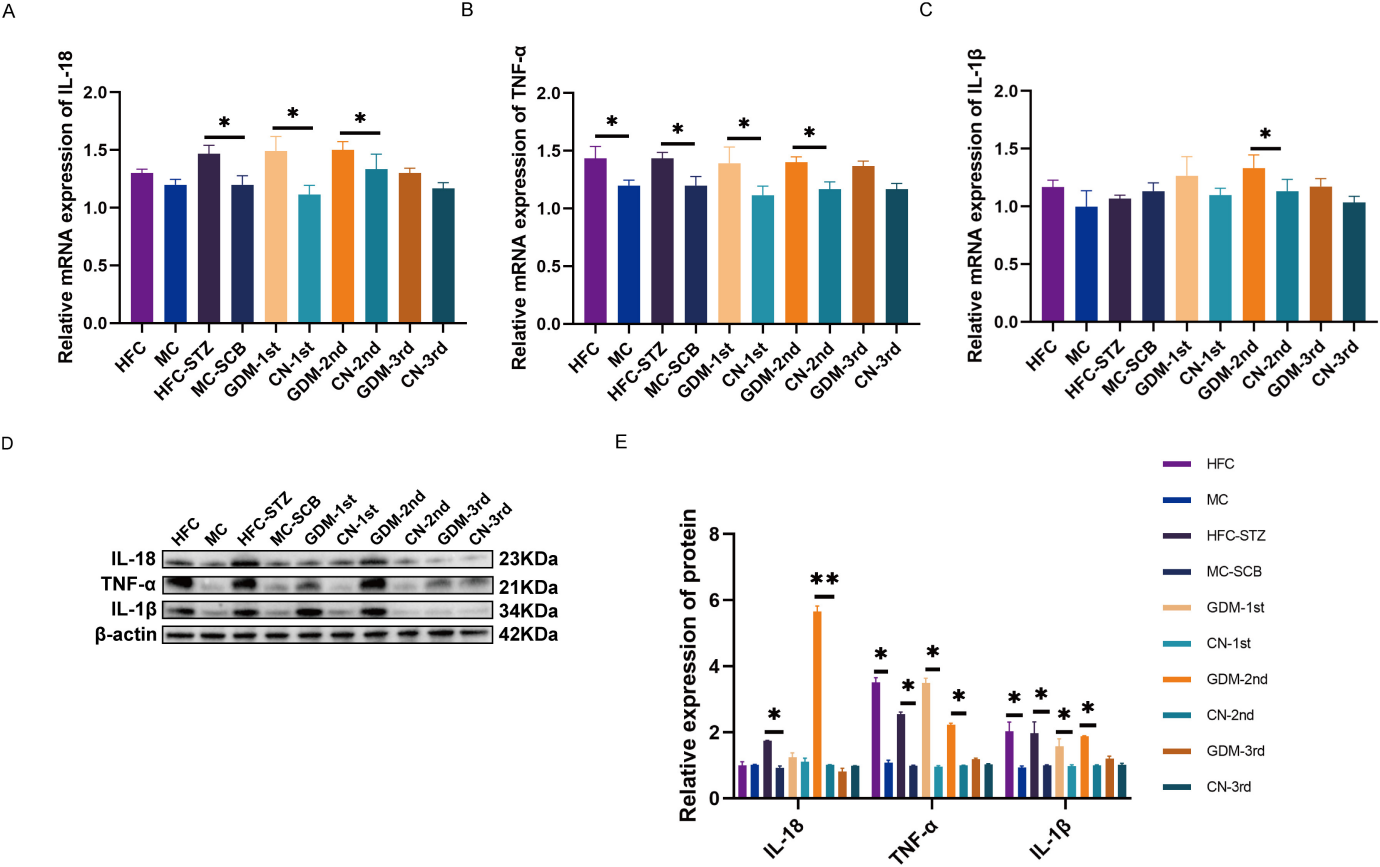
The mRNA and protein expression in the colon tissue of the five models. **(A–C)** The mRNA expression of IL-18, TNF-α, and IL-1β. **(D,E)** The protein expression of IL-18, TNF-α, and IL-1β and IL-1β. Model-1:HFC, high-fat chow; MC, maintenance chow; Model-2:HFC-STZ, high-fat chow + intraperitoneal injection of streptozotocin; MC-SCB, maintenance chow + intraperitoneal injection of sodium citrate buffer; Model-3:GDM-1st, antibiotic cocktail and fecal microbiota transplantation of the fecal in the first trimester of gestational diabetes mellitus patients; CN-1st, antibiotic cocktail and fecal microbiota transplantation of the fecal in the first trimester of health controls; Model-4:GDM-2nd, antibiotic cocktail and fecal microbiota transplantation of the fecal in the second trimester of gestational diabetes mellitus patients; CN-2nd, antibiotic cocktail and fecal microbiota transplantation of the fecal in the second trimester of health controls; Model-5: GDM-3rd, antibiotic cocktail and fecal microbiota transplantation of the fecal in the third trimester of gestational diabetes mellitus patients; CN-3rd, antibiotic cocktail and fecal microbiota transplantation of the fecal in the third trimester of health controls. Data are shown as mean ± SEM. Model-1 (*n* = 20; HFC: *n* = 10, MC: *n* = 10), Model-2 (*n* = 17; HFC-STZ: *n* = 9, MC-SCB: *n* = 8), Model-3 (*n* = 15; GDM-1st: *n* = 8, CN-1st: *n* = 7), Model-4 (*n* = 16; GDM-2nd: *n* = 8, CN-2nd: *n* = 8), Model-5 (*n* = 17; GDM-3rd: *n* = 9, CN-3rd: *n* = 8). *p < 0.05, **p < 0.01.

Next, we looked into the mRNA and protein expression of inflammatory cytokines, including IL-18, TNF-α, and IL-1β in colon tissues. Our findings revealed that in the case of modeling mice 1, 2, 3, and 4, there was an increase in the relative mRNA and protein expression of TNF-α, in comparison to the control group. The expression of IL-18 mRNA and protein expression in the colon tissues of modeling mice in model-2 and 4 were higher compared to controls. The mRNA and protein expression of IL-1β in modeling mice of model-4 were higher than those in the control mice (Figure 6).

### 3.4 Fecal microbiota from GDM patients induces gut microbial dysbiosis in mice

Based on a comprehensive analysis of blood glucose phenotypes and inflammatory factor expression across the five mouse models, we conclude that mice transplanted with fecal microbiota from second-trimester GDM patients developed a GDM-like phenotype comparable to that induced by traditional modeling methods. Therefore, we subsequently performed 16S rRNA sequencing analysis on the fecal microbiota of the first two traditional model groups (HFC and HFC-STZ) and the GDM fecal transplant model group (GDM-2nd).

**FIGURE 7:**
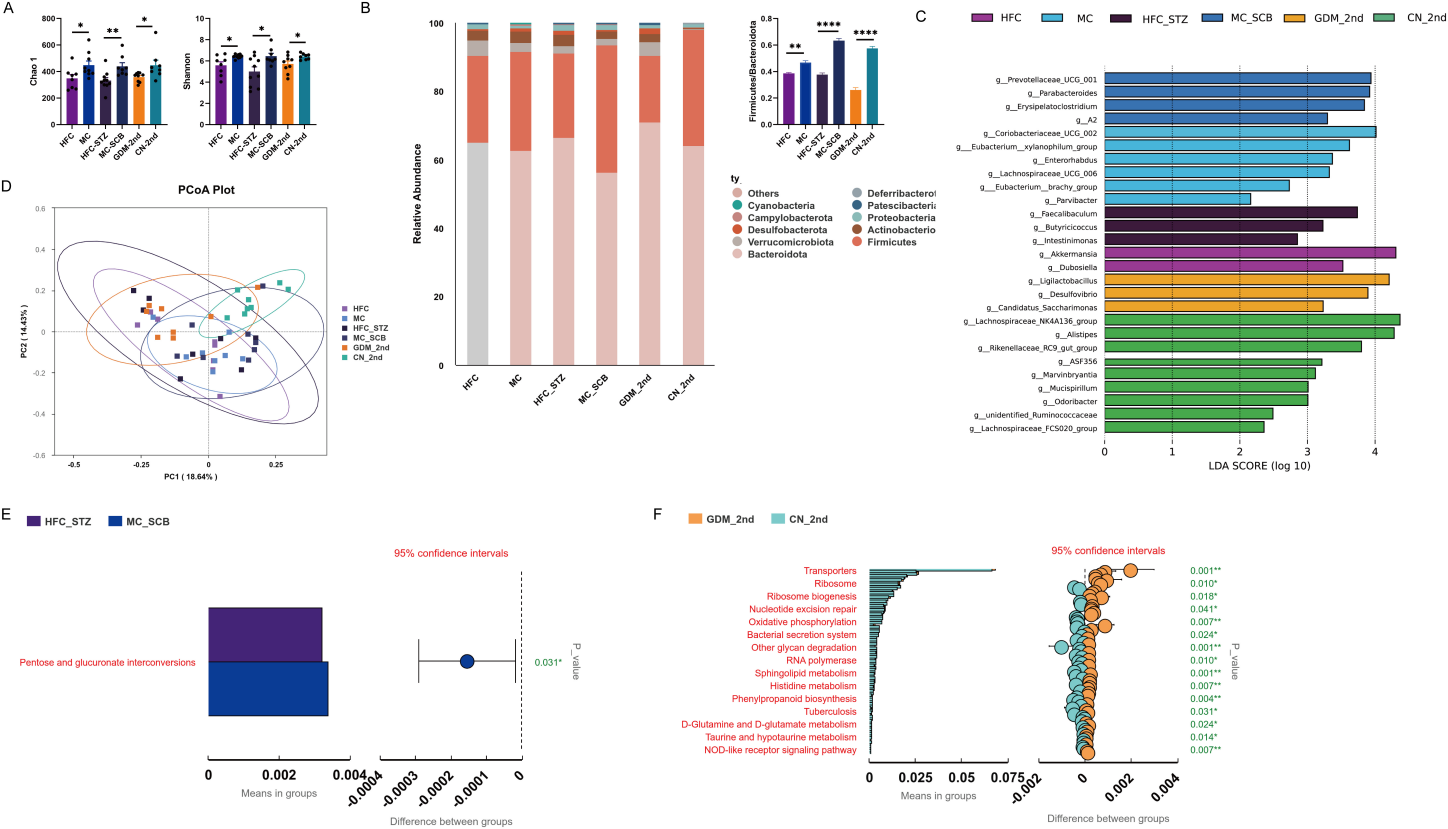
The sequencing results of 16SrRNA for the three groups of model mice. **(A)** α-diversity indices (Chao1 and Simpson). **(B)** The species abundance histograms at the phylum level and the F/B phylum bar charts for the two groups. **(C)** LEfSe analysis, presented as an LDA Score bar chart at the genus level, LDA > 2. **(D)** PCoA plot of β-diversity, calculated based on the Bray-Curtis dissimilarity metric. **(E,F)** Relative abundance of Kyoto Encyclopedia of Genes and Genomes (KEGG) pathways in the gut microbiota, predicted by Tax4fun. HFC, high-fat chow; MC, maintenance chow; HFC-STZ, high-fat chow + intraperitoneal injection of streptozotocin; MC-SCB, maintenance chow + intraperitoneal injection of sodium citrate buffer; GDM-2nd, antibiotic cocktail and fecal microbiota transplantation of the fecal in the second trimester of gestational diabetes mellitus patients; CN-2nd, antibiotic cocktail and fecal microbiota transplantation of the fecal in the second trimester of health controls. Data are shown as mean ± SEM. Model-1 (*n* = 20; HFC: *n* = 10, MC: *n* = 10), Model-2 (*n* = 17; HFC-STZ: *n* = 9, MC-SCB: *n* = 8), Model-3 (*n* = 15; GDM-1st: *n* = 8, CN-1st: *n* = 7), Model-4 (*n* = 16; GDM-2nd: *n* = 8, CN-2nd: *n* = 8), Model-5 (*n* = 17; GDM-3rd: *n* = 9, CN-3rd: *n* = 8). *p < 0.05, **p < 0.01, ****p < 0.0001.

Compared to the control groups (MC and MC-SCB), the alpha diversity indices (Chao1 and Shannon) were significantly reduced in the modeling groups (HFC and HFC-STZ). Notably, the alpha diversity indices of our fecal microbiota transplantation model mice were also significantly lower than those of the control group, indicating that transplantation of GDM microbiota similarly reduces the microbial diversity of recipient mice, thereby recapitulating the GDM phenotype (Figure 7A). The overall composition at the bacterial phylum level was similar across the different groups, primarily consisting of Firmicutes, Bacteroidota, Proteobacteria, Actinobacteria, and Verrucomicrobiota, among others. However, compared to the control groups, the Firmicutes/Bacteroidota (F/B) ratio was increased in the HFC, HFC-STZ, and GDM-2nd groups, indicating that both the classic GDM model and the GDM microbiota transplantation model induced similar patterns of microbial dysbiosis (Figure 7B).

PCoA analysis of beta diversity showed clear separation between the modeling groups and the control groups across all three models. Furthermore, samples from the GDM-2nd group highly overlapped with those from the HFC and HFC-STZ groups, demonstrating that the overall structure of the gut microbiota in mice transplanted with GDM patient feces became highly similar to that of the traditional GDM mouse models (Figure 7D).

Focusing on key changes at the genus level, we found a significant enrichment of g__Dubosiella in the HFC group, a genus associated with high-fat diet-induced obesity and inflammation (Feng et al., 2025). The HFC-STZ group showed significant enrichment of g__Faecalibaculum, a genus previously reported to be increased in obesity and diabetes models and closely related to host metabolic disorders (Ye et al., 2021). In the GDM-2nd group, g__Desulfovibrio was significantly enriched; this sulfate-reducing bacterium may damage intestinal epithelial cells (Zhou et al., 2024), and its enrichment provides strong evidence for the “pathogenicity” of the GDM microbiota. In the control groups, g__Prevotellaceae_UCG_001 was significantly enriched in the MC-SCB group, g__Eubacterium_xylanophilum_group was enriched in the MC group, and g__Lachnospiraceae_NK4A136_group was enriched in the CN-2nd group. These genera have been reported to be associated with short-chain fatty acid production and are generally considered markers of a healthy microbiota (Fusco et al., 2023; Figure 7C). Further pathway enrichment analysis revealed altered pentose and glucuronate interconversions in the HFC-STZ group compared to the MC-SCB group. Comparisons between the GDM-2nd and CN-2nd groups showed significant differences in metabolism- and inflammation-related pathways such as oxidative phosphorylation, the NOD-like receptor signaling pathway, D-glutamine and D-glutamate metabolism, and taurine and hypotaurine metabolism (Figures 7E,F). These findings suggest that both model types may develop metabolic abnormalities and pro-inflammatory potential induced by gut microbiota dysbiosis.

## 4 Discussion

Our study demonstrates that FMT from women with GDM induces excessive gestational weight gain, glucose intolerance, and insulin resistance in recipient mice. Although serum levels of certain inflammatory cytokines were elevated, no significant histopathological changes were observed in colonic tissues. 16S rRNA sequencing further confirmed that mice receiving mid-trimester GDM microbiota developed GDM-like phenotypes accompanied by gut microbial dysbiosis, characterized by reduced alpha diversity, an increased Firmicutes/Bacteroidota ratio, enrichment of pathobionts, and alterations in metabolism- and inflammation-related pathways. These features closely resemble the dysbiotic patterns observed in traditional GDM mouse models, including HFC-fed and STZ-induced hyperglycemic models. Importantly, the FMT-based GDM model recapitulates key metabolic and inflammatory aspects of GDM without using chemical or dietary induction, providing a novel and physiologically relevant tool to investigate the causative role of gut microbiota in GDM pathogenesis (Supplementary Table 4).

Excessive weight gain during pregnancy is a significant risk factor for GDM. We found that both a high-fat diet and fecal microbiota transplantation could cause mice to gain more weight during pregnancy than the control group. Clinical studies have found that excessive weight gain in the first and second trimesters of pregnant women with normal pre-pregnancy body mass index (BMI) is significantly associated with the occurrence of GDM (Brunner et al., 2015; Carreno et al., 2012). In addition, some pregnant women with GDM already have excessive weight gain at the time of diagnosis (Zheng et al., 2021). The Institute of Medicine (IOM) gestational weight gain guidelines mentioned that excessive weight gain before oral glucose tolerance test may increase the risk of GDM (National Academy of Sciences [US], 2009; Qi et al., 2020). This suggests that excessive weight gain during pregnancy is an important factor promoting the development of GDM, and high-fat diet is the culprit. Studies have revealed that a high-fat diet triggers inflammation in the hypothalamus, inducing the body to consume more calories, leading to an increased desire to eat and eventually causing weight gain and disease attack (Kim et al., 2019). In the present study, the mice in model-1 and model-2 were given a high-fat diet for 4 weeks before and during the all pregnancy, resulting in a higher weight gain compared to the mice in the normal diet group. Other animal studies have also confirmed that a diet rich in saturated fat before and during pregnancy may lead to pathological manifestations similar to human GDM in mice (Liang et al., 2010). However, unlike high-fat diet models that primarily rely on overnutrition and lipotoxicity, our FMT model reveals an independent pathway driven by gut microbiota dysbiosis: GDM-associated microbiota promotes metabolic disorders by triggering systemic inflammation and altering microbial metabolites (such as short-chain fatty acids and bile acids), as evidenced by the unique microbial composition changes observed in the GDM-FMT group. This distinction highlights that even in the absence of a high-fat diet, GDM-related gut microbiota dysbiosis alone is sufficient to drive key phenotypes, providing new insights into the heterogeneous mechanisms underlying GDM. Although this high-fat diet model has been used in many studies to understand GDM, it only partially explains its pathogenesis. GDM can occur in pregnant women with normal BMI and less weight gain during pregnancy. A study revealed that only 15.1% of GDM cases among Asians/Pacific islanders were due to obesity (Kim et al., 2012). Different races, dietary habits and pre-pregnancy weight can cause GDM differently (Fakhrul-Alam et al., 2020; Furukawa and Kobayashi, 2019). Moreover, GDM refers to abnormal glucose metabolism during pregnancy, which disappears about 1–2 weeks after delivery. But, will the high-fat diet model-induced abnormal glucose metabolism in mice disappear after delivery? It seems that this method does not fully explain the GDM’s pathophysiological mechanism. We need evidence to remains to confirm whether GDM mice induced by a high-fat diet are the best fit GDM model.

Streptozotocin is a compound that contains glucosamine-nitroso and selectively kills beta cells by being transported into cells through the glucose transporter 2 (GLUT2) (Schnedl et al., 1994). It primarily causes cell death through DNA damage, leading to necrosis and/or apoptosis (Arison et al., 1967; Murata et al., 1999; Schein et al., 1974). This method is commonly used as a model for type 1 diabetes mellitus (T1DM) to permanently destroy islet cells in mice and induce insufficient insulin secretion to mimic abnormal glucose metabolism. Pregnancy is a complex and special physiological process. With the growth and development of the fetus, the material requirements of the fetus for the mother gradually increase. In order to meet this demand, pregnant women undergo a series of physiological adaptations during pregnancy, including metabolic adaptations, mainly manifested as physiological insulin resistance (IR). With the increase of gestational age, the placenta secretes prolactin, estrogen, progesterone, human placental prolactin, human placental growth hormone, cortisol, adrenocorticotropic hormone, and suprarenal releasing hormone. Among them, human placental prolactin, human placental growth hormone, and cortisol play a major role in placental IR, which appear from the beginning of placental formation, increase with the progress of gestational age, and reach the peak in the third trimester. In order to maintain normal glucose metabolism, the number of islets β cells and insulin secretion increase significantly in pregnant women compared to non-pregnant women, and the compensatory secretion of postprandial insulin is even more pronounced (Baeyens et al., 2016; Powe et al., 2019). However, if there is insufficient secretion of insulin or the body become resistant to it, GDM can occur. It should be noted that STZ -based models are not very effective in reproducing the clinical outcomes of GDM, such as macrosomia (Gewolb et al., 1982). While STZ-based models can be useful in studying how GDM affects fetal development, they are not ideal for studying the causes of GDM.

We utilized FMT technology to transplant gut microbiota from GDM patients in their first, second, and third trimesters into ABX mouse models. The results demonstrated that mice receiving mid- and late-trimester GDM microbiota transplants exhibited elevated fasting blood glucose, impaired glucose tolerance, and insulin tolerance, along with significant alterations in insulin sensitivity and islet function indicators such as HOMA-IR and HOMA-β%, successfully replicating the core metabolic phenotype of GDM. Compared to previous studies, our experimental design did not rely on external chemical or dietary interventions. This key advantage allows the model to more closely mimic the natural progression of GDM and avoids the irreversible damage to pancreatic beta cells characteristic of STZ-induced models. The research by Liu et al. (2020) transplanted gut microbiota from GDM patients into germ-free mice and observed significantly elevated blood glucose levels 13 days post-transplantation, with early-trimester GDM microbiota also capable of inducing hyperglycemia. However, these studies did not use pregnant mouse models and thus could not assess pregnancy-specific changes (Qin et al., 2022). Employed a high-fat diet combined with FMT in pregnant mice to establish a GDM model, finding elevated blood glucose levels in GTT tests on gestation days 12 and 18, but this model incorporated both dietary and microbiota intervention factors simultaneously. In contrast, our study is the first to systematically transplant microbiota from all three trimesters of GDM patients separately and to use ABX-pretreated mice rather than germ-free mice. This approach effectively cleared the existing microbiota while better preserving the normal intestinal structure and function of the mice, overcoming limitations of germ-free mice such as thin intestinal muscular layers, enlarged cecum, and abnormal circulatory systems, thereby enhancing the physiological relevance and experimental feasibility of our model.

In terms of microbial diversity and structural changes, this study observed that GDM-FMT mice exhibited significantly reduced gut microbiota alpha diversity, markedly altered beta diversity, and community structure dysbiosis manifested as an increased Firmicutes/Bacteroidota (F/B) ratio and enrichment of conditionally pathogenic bacteria such as the genus Desulfovibrio. These findings are highly consistent with previous research results. Compared to pregnant women with normal blood glucose levels, patients with gestational diabetes showed alterations in the diversity of their gut microbiota, including both alpha and beta diversity. This imbalance led to dysbiosis, characterized by a loss of balance between beneficial and harmful bacteria, thereby triggering metabolic disorders, elevated blood glucose, and insulin resistance (Hu et al., 2023). It is noteworthy that the enrichment of Desulfovibrioobserved in our study also aligns with the results of some clinical studies (Crusell et al., 2018), suggesting that it may disrupt intestinal barrier integrity through hydrogen sulfide production, thereby promoting systemic low-grade inflammation and metabolic disorders (Villanueva-Millan et al., 2022).

Gut dysbiosis may affect metabolism through the following mechanisms: increased intestinal permeability, enhanced absorption of lipopolysaccharide (LPS), disruption of short-chain fatty acid (SCFA) production, altered conversion of primary to secondary bile acids, and increased production of bacterial metabolites such as trimethylamine N-oxide (TMAO). These changes further activate inflammatory and autoimmune pathways, promote molecular mimicry of self-antigens, and impair insulin signaling (de Mendonça et al., 2022; Meijnikman et al., 2018). Studies have confirmed that hyperglycemia can compromise intestinal barrier function by affecting the proliferation, maturation, and mucus secretion of goblet cells, thereby allowing harmful bacteria and LPS to cross the barrier (Zhang et al., 2021). LPS, in turn, binds to Toll-like receptors on the surface of intestinal epithelial cells, recruiting immune cells in the gut and releasing pro-inflammatory factors such as IL-1 and IFN-γ, disrupting internal homeostasis (Lv et al., 2020), and triggering immune responses and chronic inflammation. These factors collectively lead to further dysbiosis of the gut microbiota (Buford, 2017). In this study, following the transplantation of gut microbiota from GDM patients into ABX mice, we observed significantly elevated levels of multiple inflammatory factors (such as TNF-α, IL-1β, IL-18, resistin, and GDF-15) in both the serum and colonic tissues of the recipient mice. However, no obvious pathological damage or inflammatory cell infiltration was detected in the colon tissue, indicating that the inflammatory state remains at a “subclinical” stage. This finding is consistent with the clinical features of GDM, where patients typically do not exhibit significant intestinal inflammation or elevated inflammatory cell counts in the blood (Ozuguz et al., 2011; Pöyhönen-Alho et al., 2011; Volpe et al., 2007). Resistin is a polypeptide primarily secreted by adipocytes that contributes to insulin resistance by inhibiting glucose uptake. Resistin can directly bind to Toll-like receptor 4 (TLR-4) and adenylyl cyclase-associated protein 1 (CAP1) receptors, initiating pro-inflammatory responses in both humans and mice (Benomar et al., 2013; Tarkowski et al., 2010). Consistent with previous studies, we also detected increased resistin levels in the serum of GDM-2nd FMT mice, along with elevated levels of IL-18, TNF-α, and IL-1β in the colonic tissues of this group. This may be attributed to increased weight gain during pregnancy in these mice, leading to enhanced resistin secretion by adipocytes and elevated levels of pro-inflammatory cytokines TNF-α and IL-1β. GDF-15 is involved in inflammation, cell repair, and tissue growth after injury. It is present in the placenta and fetal membranes (Fejzo et al., 2018), and its serum concentration is elevated in GDM patients (Yakut et al., 2021). GDF-15 is also positively correlated with fasting blood glucose, 1-h postprandial blood glucose, 2-h postprandial blood glucose, glycated hemoglobin, and the area under the curve for blood glucose in the oral glucose tolerance test (OGTT) (Tang et al., 2019). The exact mechanism of GDF-15 in patients with glucose metabolism disorders remains unclear. MMP-9 is a member of the matrix metalloproteinase (MMPs) family and participates in angiogenesis, inflammation, wound repair, growth, and development (Ray and Stetler-Stevenson, 1994). Most studies have found associations between MMPs and various physiological processes, such as ovulation (Liu et al., 1998), embryo implantation (Alexander et al., 1996), embryonic development (Chin and Werb, 1997), and gestational trophoblastic disease (Okamoto et al., 2002). MMP-9 was found to be increased in the placenta of diabetic rats during the second trimester of pregnancy (Pustovrh et al., 2005). The elevated serum MMP-9 levels in GDM-2nd FMT mice in our study may also originate from the placenta, although further experiments are needed to confirm this.

The strengths of our study include the following aspects. First, the use of an ABX mouse model better simulates the physiological environment of host-microbe interactions. Second, the FMT protocol covering both pre-pregnancy and pregnancy stages enhances the authenticity of disease progression modeling. Third, direct comparison with established HFD and STZ models validates the translational value of our model. However, several limitations should be noted. Firstly, the lack of baseline microbial composition analysis of donor feces prevents the assessment of the impact of specific bacterial strains onthe phenotypes. Future studies should stratify donors based on key clinical features such as, dietary patterns, BMI, or specific glycemic parameters, and integrate metagenomics to identify key microbial signatures. Secondly, although FMT successfully induced phenotypes, the correlationbetween microbial changes and metabolic abnormalities still requires mechanistic validation, for instance, through colonization experiments in germ-free models. We recommend that future research adopt adopting multi-omics approaches, such as non-targeted metabolomics, to analyze serum and fecal samples to identify key molecules mediating the metabolic effects. Furthermore, evaluating fetal outcomes is essential to fully reveal the intergenerational impact of GDM. Lastly, we emphasize that this model is designed to explore the mechanisms of GDM rather than for direct clinical translation. While FMT is not currently recommended for pregnant women, the microbial targets identified could inspire safer interventional strategies. Future mechanistic experiments are needed to further validate the causal chain. In summary, our study, utilizing an FMT model, confirms the significant role of gut microbiota in GDM and provides a theoretical foundation for future targeted microbial interventions.

## 5 Conclusion

Our study demonstrates that FMT from second- and third-trimester GDM patients recapitulates key clinical features of GDM—including glucose intolerance, insulin resistance, and excessive weight gain—in murine models. 16S rRNA sequencing revealed that GDM-FMT induced gut microbial dysbiosis characterized by reduced alpha diversity, an elevated Firmicutes/Bacteroidota ratio, and enrichment of pathobionts, closely resembling dysbiotic patterns in traditional HFC and HFC-STZ models. This microbiota disruption triggered subclinical gut inflammation and systemic metabolic-inflammatory dysregulation, despite the absence of overt histopathological damage. These findings underscore the trimester-specific influence of GDM microbiota and highlight its role not only as a diagnostic biomarker but also as a therapeutic target for intervening in maternal metabolic dysfunction. Future studies should focus on identifying causal microbial taxa and their mechanistic pathways to enable precision microbiome-based therapies.

## Data Availability

The original contributions presented in the study are included in the article/Supplementary material, further inquiries can be directed to the corresponding author.
