# Supplementary material for: Fecal microbiota transplantation from gestational diabetes mellitus patients induces glucose intolerance and subclinical inflammation in mice
Source: Front Microbiol. 2026 Jan 28;16:1723816. doi: 10.3389/fmicb.2025.1723816 (PMC12893721; doi:10.3389/fmicb.2025.1723816)
Supplement: Supplementary file 1 [file Supplementary_file_1.docx]

Supplementary Materials

**Supplementary Table 1. Composition of chow**

| Ingredient | [Maintenance](javascript:;) chow |  | High-fat chow |  |
| --- | --- | --- | --- | --- |
|  | gm | kcal | gm | kcal |
| Casein, 80Mesh | 0 | 0 | 200 | 800 |
| Casein, 30Mesh | 140 | 560 | 0 | 0 |
| L-Cystine | 1.8 | 7.2 | 3 | 12 |
| Corn Starch | 495.692 | 1983 | 72.8 | 291 |
| Maltodextrin 10 | 125 | 500 | 100 | 400 |
| Sucrose | 100 | 400 | 172.8 | 691.2 |
| Cellulose | 50 | 0 | 50 | 0 |
| Soybean Oil | 40 | 360 | 25 | 225 |
| t-Butylhydroquinone | 0.008 | 0 | 0 | 0 |
| Mineral Mix | 35 | 0 | 10 | 0 |
| Vitamin Mix | 10 | 40 | 10 | 40 |
| Choline Bitartrate | 2.5 | 0 | 2 | 0 |
| Lard | 0 | 0 | 177.5 | 1598 |
| Dicalcium Phosphate | 0 | 0 | 13 | 0 |
| Calcium Carbonate | 0 | 0 | 5.5 | 0 |
| Potassium Citrate | 0 | 0 | 16.5 | 0 |
| Total | 1000 | 3850.2 | 858.1 | 4057.2 |

**Supplementary Table 2. The Basic Characteristics of Fecal Donors**

| Maternal and neonatal characteristics | Control | | | | GDM | | | | P^a^ value | P^b^ value | P^c^ value | P^d^ value |
| --- | --- | --- | --- | --- | --- | --- | --- | --- | --- | --- | --- | --- |
|  | All (N=42) | First trimester (N=14) | Sencond trimester (N=14) | Third trimester (N=14) | All (N=42) | First trimester (N=14) | Sencond trimester (N=14) | Third trimester (N=14) |  |  |  |  |
| Age, years | 34.095±3.850 | 36.286±3.429 | 33.929±3.430 | 32.071±3.689 | 33.905±3.862 | 35.428±3.005 | 33.000±4.261 | 33.286±4.008 | 0.488 | 0.531 | 0.412 | 0.821 |
| Gravidity, 1st | 6 (14.286%) | 0 (0%) | 0 (0%) | 6 (42.857%) | 14 (33.333%) | 3 (21.429%) | 6 (42.857%) | 5 (35.714%) | 0.072 | 0.007* | 0.704 | 0.042* |
| Parity, 1st | 11 (26.190%) | 0 (0%) | 1 (7.143%) | 10 (71.429%) | 19 (45.238%) | 5 (35.714%) | 8 (57.143%) | 6 (42.857%) | 0.097 | 0.005* | 0.134 | 0.070 |
| Height, cm | 161.143±4.502 | 160.929±5.980 | 160.857±3.959 | 161.643±3.478 | 161.810±5.134 | 163.714±4.565 | 160.643±5.108 | 161.071±5.498 | 0.178 | 0.902 | 0.745 | 0.529 |
| Prepregnancy weight, kg | 55.750±6.892 | 58.714±7.498 | 56.286±7.332 | 52.250±4.108 | 60.393±11.135 | 65.678±7.893 | 58.714±12.344 | 56.786±11.369 | 0.024* | 0.532 | 0.172 | 0.024* |
| Prepregnancy BMI, kg/cm2 | 21.494±2.722 | 22.680±2.678 | 21.794±3.093 | 20.009±1.622 | 23.029±3.951 | 24.573±3.478 | 22.668±4.169 | 21.845±3.944 | 0.119 | 0.534 | 0.119 | 0.041* |
| Delivery weight, kg | 70.088±8.049 | 72.000±8.988 | 70.750±8.106 | 67.514±6.806 | 71.412±9.600 | 76.300±6.423 | 69.936±11.185 | 68.000±9.148 | 0.157 | 0.827 | 0.875 | 0.495 |
| GWG, kg | 14.338±4.940 | 13.285±4.103 | 14.464±4.774 | 15.264±5.935 | 11.019±5.573 | 10.621±4.842 | 11.221±5.046 | 11.214±6.985 | 0.128 | 0.092 | 0.110 | 0.005* |
| Cesarean section | 13 (30.952%) | 5 (35.714%) | 6 (42.857% | 2 (14.286%) | 16 (38.095%) | 6 (42.857%) | 6 (42.857% | 4 (28.571%) | 0.704 | 1.000 | 0.366 | 0.494 |
| Neonatal male sex | 24 (57.143%) | 9 (64.286%) | 10 (71.429%) | 5 (35.714%) | 29 (69.048%) | 10 (71.429%) | 9 (64.286%) | 10 (71.429%) | 0.691 | 0.691 | 0.063 | 0.261 |
| Birth weight, g | 3407.738±342.249 | 3474.28±414.543 | 3415.714±223.175 | 3333.214±370.071 | 3342.143±388.733 | 3526.786±288.401 | 3307.500±498.339 | 3192.143±287.808 | 0.700 | 0.465 | 0.270 | 0.414 |
| Gestational age, weeks | 39 (38-39) | 39 (38-40) | 39 (38-39) | 39 (38-40) | 39 (38-39) | 39 (38-39) | 39 (38-40) | 39 (38-39) | 0.845 | 0.338 | 0.307 | 0.841 |
| Macrosomia | 3 (7.143%) | 2 (14.286%) | 0 (0%) | 1 (7.143%) | 1 (2.381%) | 0(0%) | 1 (7.143%) | 0 (0%) | 0.150 | 0.317 | 0.317 | 0.308 |

P^a^: Comparison between the control group and the GDM group in the first trimester; P^b^: Comparison between the control group and the GDM group in the second trimester; P^c^: Comparison between the control group and the GDM group in the third trimester. P^d^: Comparison of the entire population between the control group and the GDM group. GDM, gestational diabetes mellitus; BMI, body mass index; GWG, gestational weight gain.*P<0.05.

**Supplementary Table 3. Primer sequences**

| Primer | Sequences (5'->3') | |
| --- | --- | --- |
|  | Forward | Reverse |
| IL-18 | GACAGCCTGTGTTCGAGGAT | GGTGGATCCATTTCCTCAAAGG |
| TNF-α | GGCCTCCCTCTCATCAGTTC | GGTGGTTTGCTACGACGTG |
| IL-1β | AAGCTCTCCACCTCAATGGAC | CTTGGGATCCACACTCTCCAGC |
| GAPDH | TCAGGAGAGTGTTTCCTCGTC | GCCGTTGAATTTGCCGTGAG |

**Supplementary Table 4. Comparison Between GDM Models**

| Parameter​ | GDM-FMT Model​ | | | | | | | | | HFC​ | MC | P value | HFC-STZ ​ | MC-SCB | P value |
| --- | --- | --- | --- | --- | --- | --- | --- | --- | --- | --- | --- | --- | --- | --- | --- |
|  | GDM-1st | CN-1st | P value | GDM-2nd | CN-2nd | P value | GDM-3rd | CN-3rd | P value |  |  |  |  |  |  |
| Gestational weight gain (g) | 7.175 (7.070, 7.230) | 7.080 (7.000, 7.220) | 0.0207 | 8.940 (8.670, 9.445) | 7.220 (7.028, 7.195) | 0.013* | 8.485 (8.250, 8.645) | 7.105 (7.008, 7.218) | 0.004* | 9.605 (8.000, 11.125) | 7.215 (7.083, 7.311) | 0.031* | 9.150 (8.200, 9.650) | 7.113 (7.063, 7.411) | 0.002* |
| Vaginal Plug Rate (%) | 24 (80.00%) | 23 (76.67%) | 0.351 | 25 (83.33%) | 24 (80.00%) | 0.224 | 25 (83.33%) | 25 (83.33%) | 0.632 | 29 (96.67%) | 24 (80.00%) | 0.024* | 28 (93.33%) | 23 (76.67%) | 0.031* |
| Conception rate (%) | 8  (33.33%) | 7 (30.43%) | 0.671 | 8 (32.00%) | 8 (33.33%) | 0.551 | 9 (36.00%) | 8 (32.00%) | 0.433 | 10 (34.48%) | 10 (41.67%) | 0.061 | 9 (32.14%) | 8 (34.78%) | 0.123 |
| Glucose tolerance test (AUC, mg/dL×min) | 1381.50 (1356.75, 1397.00) | 1365.00 (1326.76, 1396.41) | 0.332 | 1567.53 (1509.82, 1595.25) | 1371.11 (1311.65, 1412.56) | 0.043* | 1513.65 (1427.55, 1587.00) | 1376.32 (1312.58, 1400.61) | 0.041* | 1626.00 (1569.14, 1659.06) | 1367.98 (1288.11, 1405.79) | 0.033* | 1670.43 (1576.87, 1699.01) | 1382.15 (1326.01, 1396.58) | 0.015* |
| Insulin tolerance test (AUC，mg/dL×min) | 365.91 (353.75, 381.00) | 331.96 (424.65, 365.12) | 0.051 | 550.51 (472.75, 582.54) | 367.12 (361.05, 388.15) | 0.013* | 551.09 (477.14, 574.15) | 345.83 (332.97, 398.71) | 0.042* | 566.98 (520.18, 588.11) | 371.03 (336.58, 381.09) | 0.026* | 512.98 (498.16, 588.09) | 370.16 (366.51, 387.91) | 0.026* |
| Fasting blood glucose (mg/dL) | 115.50±5.11 | 109.34±2.31 | 0.067 | 148.35±4.28 | 98.65±3.08 | 0.033* | 121.04±3.56 | 106.87±4.76 | 0.078 | 169.64±2.98 | 98.78±3.91 | 0.011* | 173.28±3.19 | 101，63±2.66 | 0.003* |
| Fasting insulin (μIU/mL) | 19.26±2.03 | 16.67±1.11 | 0.079 | 22.37±2.21 | 15.74±2.00 | 0.042* | 18.79±2.11 | 16.04±1.43 | 0.077 | 23.73±2.04 | 17.88±2.22 | 0.033* | 9.14±1.29 | 16.83±1.79 | 0.007* |
| HOMA-β% | 125.35±6.11 | 166.746±5.98 | 0.064 | 89.14±4.78 | 164.98±6.18 | 0.034* | 90.54±5.98 | 168.99±6.07 | 0.077 | 75.98±5.87 | 169.45±7.12 | 0.012* | 34.25±6.12 | 171.24±5.78 | 0.001* |
| HOMA-IR | 5.15±1.15 | 3.76±1.08 | 0.113 | 6.55±2.06 | 3.88±1.78 | 0.005* | 5.78±2.00 | 4.63±1.66 | 0.067 | 7.22±1.77 | 3.86±1.75 | 0.004* | 6.95±1.17 | 5.33±1.27 | 0.003* |
| Elevated inflammatory markers in serum (FDR<0.05) | GDF-15,IL-1β | None | <0.01*, <0.01* | Resistin,  MMP-9 | None | 0.04*, 0.04* | None | None | **_** | None | None | **_** | MMP-9 | None | <0.01* |
| Length of colon | 7.840±0.789 | 7.841±1.107 | 0.898 | 7.311±1.012 | 7.469±1.211 | 0.290 | 7.780±1.062 | 7.906±1.112 | 0.200 | 7.463±0.971 | 7.768±0.893 | 0.354 | 7.557±0.878 | 7.399±0.911 | 0.139 |
| H&E staining | No significant inflammatory phenotype was observed in any group of mice. | | | | | | | | | | | | | | |
| Upregulation of mRNA | IL-18, TNF-α | None | 0.004*,0.021* | L-18, TNF-α, IL-1β | None | 0.011*, 0.041* | None | None | None | TNF-α | None | 0.042* | IL-18, TNF-α | None | 0.011*,0.036* |
| Upregulation of protein | TNF-α, IL-1β | None | 0.042*, 0.026* | IL-18, TNF-α, IL-1β | None | 0.003*, 0.025*, 0.042* | None | None | **_** | TNF-α, IL-1β | None | 0.033*, 0.042* | IL-18, TNF-α, IL-1β | None | 0.037*, 0.042*, 0.004* |
| 16SrRNA (LEfSe analysis) | **_** | **_** | _ | g__Ligilactobacillus,  g__Desulfovibrio,  g__Candidatus_Saccharimonas | g__Lachnospiraceae_NK4A136_group,  g__Alistipes,  g__Rikenellaceae_RC9_gut_group,  g__ASF356,  g__Marvinbryantia,  g__Mucispirillum,  g__Odoribacter,  g__unidentified_Ruminococcaceae,  g__Lachnospiraceae_FCS020_group | _ | _ | _ | _ | g_Akkermansia,  g_Dubosiella | g__Coriobacteriaceae_UCG_002,  g__Eubacterium_xylanophilum_group,  g__Enterorhabdus,  g__Lachnospiraceae_UCG_006,  g__Eubacterium_brachy_group,  g__Parvibacter | _ | g__Faecalibaculum,  g__Butyricicoccus, g__Intestinimonas | g_Prevotellaceae_UCG_001,  g_Parabacteroides,  g_Erysipelatoclostridium,  g_A2 | **_** |

**Supplementary Figure 1. Full membrane images for Western blot**


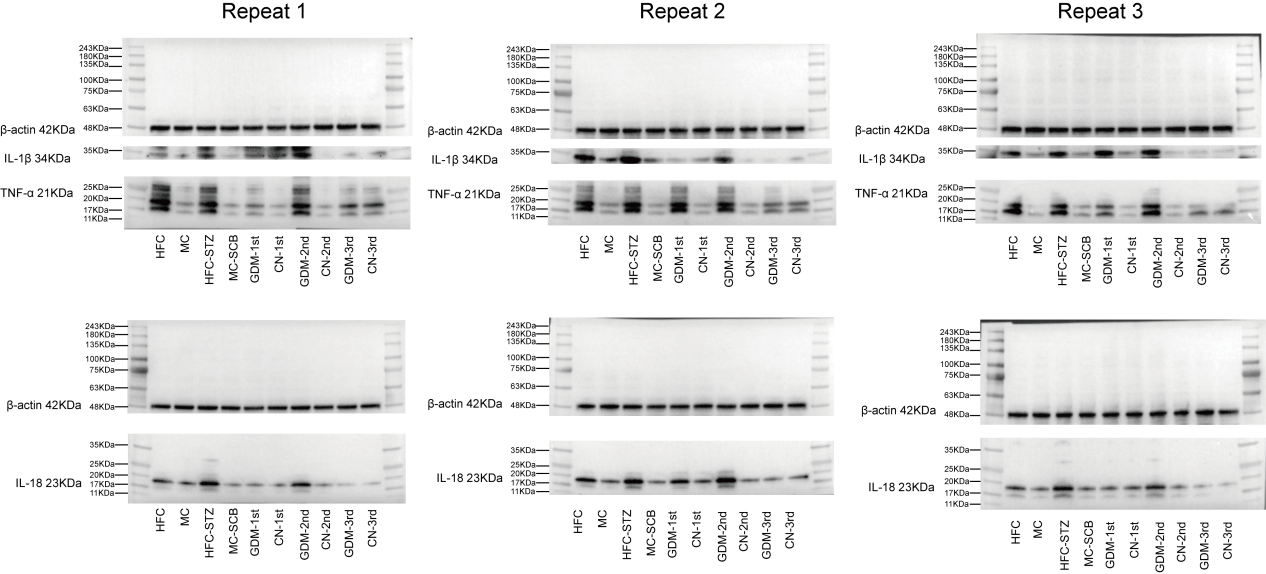


**Supplementary Method S1:**

**Sample Size Calculation**

**Justification Using the Resource Equation Method[1]**

This method is well-established in pre-clinical animal studies, particularly in complex multi-group designs where effect sizes for specific outcomes may not be known a priori, making an a priori power analysis challenging. The core principle of this method is to ensure an adequate "Error Degrees of Freedom" (E) in the subsequent statistical analysis of variance (ANOVA), which should ideally be between 10 and 20 to ensure the robustness of the F-test . The formula is E = N - T, where N is the total number of experimental units (animals), and T is the total number of experimental treatment groups. Our study involved 10 distinct treatment groups. To achieve an E value within the recommended range:

For E ≈ 10: N = E + T = 10 + 10 = 20 animals in total. This translates to n=2 animals per group.

For E ≈ 20: N = 20 + 10 = 30 animals in total. This translates to n=3 animals per group.

However, this initial calculation only meets the minimal statistical threshold. Crucially, we had to account for the specific biological context of our study, which uses pregnant mice. Based on our pre-experimental data and colony records, the mating success (pregnancy rate) of C57BL/6J mice in our facility is approximately 30-50%. Therefore, to ensure we would have a sufficient number of pregnantmice for the final analysis in each of the 10 groups, we needed to significantly increase the initial number of animals allocated to mating.

Conservative Calculation: To ensure we had at least n=3 pregnant miceper group (N=30 pregnant mice total), we needed to account for a 30% pregnancy rate. Thus, the total number of mice allocated to mating was 30 / 0.30 = 100 mice.

Target Calculation: To aim for a more robust n=6-10 pregnant miceper group, we allocated a total of approximately 300 mice to mating, which is a conservative and common practice in studies involving pregnant animal models to buffer against variability in pregnancy rates and other potential attrition.

Consequently, the final sample size of 5-6 mice per group reported in our study represents the number of pregnant micethat successfully completed the protocol in each group. This sample size is not only justified by the Resource Equation Method but is also well within or even exceeds the range commonly reported in the literature for similar metabolic phenotyping studies in mice. We are confident that this sample size provides adequate power to detect significant differences in outcomes such as glucose AUC and inflammatory markers.

**Randomization, Allocation Concealment, and Blinding**

**Randomization**: Following acclimatization, mice were assigned a unique identification number. They were then randomly assigned to one of the 10 experimental groups using a computer-generated random number sequence. This was performed by a researcher not involved in the subsequent procedures.

**Allocation Concealment**: The group allocation list was kept securely by the individual who performed the randomization and was not disclosed to the investigators responsible for the daily interventions (e.g., FMT gavage), metabolic phenotyping (e.g., GTT), and histological assessments until after the data analysis was completed.

**Blinding**: The investigators performing the key outcome measurements, including glucose tolerance tests, serum inflammatory marker assays, and histology scoring, were blinded to the group assignments. The samples were labeled only with the unique animal identification numbers to prevent bias during data collection and analysis.

**Supplementary Method S2:**

**Detailed 16S rRNA Gene Sequencing and Bioinformatic Analysis Protocol**

1. **Sample Processing and DNA Extraction**​

Frozen fecal samples (approximately 200 mg) were thawed on ice and homogenized. Genomic DNA was extracted using the Tiangen Fecal Genomic DNA Extraction Kit (DP712, Tiangen Biotech, China) according to the manufacturer's instructions. The extraction protocol included:

1. Mechanical lysis with bead beating (0.1 mm glass beads) at 6.5 m/s for 45 seconds using a FastPrep-24 instrument;
2. Incubation with proteinase K at 56°C for 30 minutes;
3. Binding to silica membrane columns;
4. Two wash steps with AW1 and AW2 buffers;
5. Elution in 100 μL EB buffer.

DNA concentration was quantified using a Qubit 4.0 Fluorometer (Thermo Fisher Scientific), and purity was assessed by NanoDrop 2000 (A260/280 ratio >1.8 required for inclusion).

1. **Library Preparation and Sequencing​**

The V3-V4 hypervariable regions (approximately 460 bp) of the bacterial 16S rRNA gene were amplified using primers 341F (5′-CCTAYGGGRBGCASCAG-3′) and 806R (5′-GGACTACNNGGGTATCTAAT-3′). PCR reactions contained 15 μL Phusion High-Fidelity PCR Master Mix (2X), 0.2 μM of each primer, and 10 ng template DNA in a 30 μL total volume. Thermal cycling conditions were: 98°C for 1 min; 30 cycles of 98°C for 10 s, 50°C for 30 s, 72°C for 30 s; final extension at 72°C for 5 min. Amplicons were purified with AMPure XP beads (0.8X ratio), quantified by Qubit, and size-verified on a Bioanalyzer 2100. Libraries were prepared using the NEB Next Ultra DNA Library Prep Kit with dual indexing and sequenced on an Illumina NovaSeq 6000 platform (2×250 bp paired-end; average 100,000 raw reads per sample).

1. **Bioinformatic Processing Pipeline​**
   1. **Data Preprocessing​**

Raw demultiplexed reads were processed in QIIME2 (v2021.4). Adapters and primers were removed using cutadapt (v3.4). Paired-end reads were merged using FLASH (v1.2.11) with a minimum overlap of 15 bp and maximum overlap of 250 bp. Quality filtering was performed with fastp (v0.23.1) using parameters: -q 20 -u 50 -n 0 -l 150. Chimeric sequences were identified and removed using vsearch (v2.16.0) against the SILVA 138 database in reference-based mode.

- 1. **ASV Analysis**​

Denoising was performed using DADA2 with truncation parameters: --p-trunc-len-f 240 --p-trunc-len-r 200. Taxonomic assignment was conducted using a naive Bayes classifier trained on the SILVA 138 database. Mitochondrial and chloroplast sequences were removed. The ASV table was rarefied to 30,000 sequences per sample for downstream diversity analyses.

- 1. **Diversity Analysis​**

Alpha diversity was assessed using the Chao1 richness index and Shannon diversity index. Beta diversity was calculated based on Bray-Curtis dissimilarity. Statistical significance of group differences was evaluated using PERMANOVA with 999 permutations (p<0.05 considered significant). Community composition differences were visualized through principal coordinates analysis (PCoA).

- 1. **Differential Abundance Analysis**​

Linear discriminant analysis effect size (LEfSe) was applied to identify differentially abundant taxa, using a logarithmic LDA score threshold of >2.0 and a p-value <0.05 (Kruskal-Wallis test). Additional validation was performed using MetagenomeSeq with a zero-inflated Gaussian model and ANCOM-BC.

- 1. **Functional Prediction​**

Functional profiling of the microbiota was inferred from 16S rRNA gene data using Tax4Fun, which maps ASVs to KEGG orthology groups to predict metabolic pathways and functional capabilities.

1. **Quality Control Measures​**

Negative controls (extraction and PCR blanks) were included in each batch, requiring <1000 sequences. Samples were included only if they yielded >20,000 high-quality reads and demonstrated >80% Good's coverage. Technical replicates showed >95% reproducibility in community composition analysis.

**References**

[1] Charan J, Kantharia ND. How to calculate sample size in animal studies? J Pharmacol Pharmacother 2013;4(4):303-6.
